# Advancing the Potential of *Polyscias fruticosa* as a Source of Bioactive Compounds: Biotechnological and Pharmacological Perspectives

**DOI:** 10.3390/molecules30173460

**Published:** 2025-08-22

**Authors:** Anita A. Śliwińska, Karolina Tomiczak

**Affiliations:** 1 Department of Pharmaceutical Biology and Medicinal Plant Biotechnology, Faculty of Pharmacy, Medical University of Warsaw, 1 Banacha St., 02-097 Warsaw, Poland; 2 Plant Breeding and Acclimatization Institute—National Research Institute, Radzików, 05-870 Błonie, Poland; k.tomiczak@ihar.edu.pl

**Keywords:** *Polyscias fruticosa*, in vitro propagation, triterpenoid saponins, flavonoids, pharmacological potential, biotechnological applications, phytoremediation

## Abstract

*Polyscias fruticosa* (L.) Harms, a Southeast Asian medicinal plant of the Araliaceae family, has gained increasing attention due to its rich phytochemical profile and potential pharmacological applications. This review provides an up-to-date synthesis of biotechnological strategies and chemical investigations related to this species. In vitro propagation methods, including somatic embryogenesis, adventitious root, and cell suspension cultures, are discussed with emphasis on elicitation and bioreactor systems to enhance the production of secondary metabolites. Phytochemical analyses using gas chromatography–mass spectrometry (GC-MS), high-performance liquid chromatography (HPLC), and nuclear magnetic resonance (NMR) have identified over 120 metabolites, including triterpenoid saponins, polyphenols, sterols, volatile terpenoids, polyacetylenes, and fatty acids. Several compounds, such as tocopherols, conjugated linoleic acids, and alismol, were identified for the first time in the genus. These constituents exhibit antioxidant, anti-inflammatory, antimicrobial, antidiabetic, anticancer, and neuroprotective activities, with selected saponins (e.g., chikusetsusaponin IVa, *Polyscias fruticosa* saponin [PFS], zingibroside R1) showing confirmed molecular mechanisms of action. The combination of biotechnological tools with phytochemical and pharmacological evaluation supports *P. fruticosa* as a promising candidate for further functional, therapeutic, and nutraceutical development. This review also identifies knowledge gaps related to compound characterization and mechanistic studies, suggesting future directions for interdisciplinary research.

## 1. Introduction

*Polyscias fruticosa* (L.) Harms, commonly referred to as Ming aralia or “Vietnamese ginseng”, is an important medicinal plant of the Araliaceae family, native to Southeast Asia and widely cultivated across tropical and subtropical regions [1,2]. It has a longstanding role in traditional medicine systems in Vietnam, the Philippines, and Indonesia, where it is used to treat a broad spectrum of conditions, including fatigue, asthma, metabolic disorders, inflammation, and neurodegenerative diseases [2,3,4]. These ethnomedical applications are consistent with the plant’s chemically diverse profile. Recent pharmacological findings also indicate similarities to *Panax ginseng*, particularly with regard to adaptogenic and immunomodulatory properties.

Phytochemical investigations have identified a wide array of bioactive constituents in *P. fruticosa*, including triterpenoid saponins, flavonoids and phenolic acids, sterols, essential oils, polyacetylenes, and fatty acids [3,5,6,7,8,9,10,11]. These structurally diverse compounds have been associated with a broad spectrum of biological activities, such as anti-inflammatory, antioxidant, antidiabetic, antimicrobial, antiasthmatic, antitussive, anti-osteoclastogenic, and neuroprotective effects [10,11,12,13,14,15,16,17,18,19,20,21]. Due to their structural and functional similarity to ginsenosides, these saponins have garnered increasing interest as potential functional analogs to those found in *Panax* species, supporting their use in both pharmaceutical and nutraceutical applications [16]. However, the broader utilization of *P. fruticosa* is hindered by slow vegetative growth, seasonal variation, and low natural yields of pharmacologically active metabolites [22]. To overcome these limitations, several in vitro culture techniques have been employed, including micropropagation, somatic embryogenesis, callus and suspension cultures, and adventitious root formation [22,23,24,25]. These biotechnological strategies not only enable sustainable biomass production but also enhance the yield of targeted secondary metabolites. For instance, elicitation with compounds such as jasmonic acid and chitosan has been shown to significantly increase flavonoid and saponin accumulation in vitro [26,27], offering a platform for the metabolic enhancement of high-value phytochemicals.

Despite significant research progress, no comprehensive review has yet addressed the multifaceted properties of *Polyscias fruticosa* as a medicinal plant. Therefore, the aim of this critical review is to integrate current knowledge on the following: (i) biotechnological methods enhancing biomass and secondary metabolite production; (ii) phytochemical diversity, with particular emphasis on compound classification and structural characteristics; and (iii) pharmacological activities supported by mechanistic evidence. In addition, this review provides the authors’ perspective on research gaps, the biosynthetic plausibility of identified compounds, and directions for future studies. By presenting *P. fruticosa* as a plant species with high research potential in the fields of biotechnology and pharmacology, this work aims to highlight its relevance for further investigations into natural products and plant-derived therapeutic strategies.

## 2. Materials and Methods

The literature for this review was collected through a comprehensive search of scientific databases including PubMed, Scopus, Web of Science, and Google Scholar. Keywords such as “*Polyscias fruticosa*,” “bioactive compounds,” “in vitro culture,” “pharmacological activity,” and “biotechnology” were used. Studies published in English between 2000 and 2024 were prioritized; however, earlier foundational works and historically significant publications were also included when relevant, particularly those providing essential methodological background or pioneering data.

The selection criteria encompassed relevance to the topic, experimental rigor, and contribution to understanding the biotechnological and pharmacological potential of *Polyscias fruticosa*. Priority was given to peer-reviewed original research articles, reviews, and phytochemical studies reporting detailed extraction methods, compound identification (e.g., by GC-MS, HPLC, NMR), and bioactivity assessments.

Duplicate reports and publications lacking primary data or methodological transparency were excluded. Data were organized thematically and interpreted critically to identify key findings, research gaps, and emerging perspectives in the field.

## 3. Morphology, Systematics, Distribution, and Ethnobotanical Relevance of *Polyscias fruticosa* (L.) Harms

*Polyscias fruticosa* is a perennial, glabrous shrub or undershrub in the Araliaceae family, characterized by a woody, sparsely to moderately branched stem and a distinctive crown of finely dissected, compound leaves. The species is widely cultivated in tropical and subtropical regions of Asia, the Pacific Islands, Africa, and the Caribbean, primarily for its ornamental and medicinal properties. It typically grows to a height of 1.5 to 4 m, depending on environmental conditions and cultivation practices [1,2].

**Figure 1:**
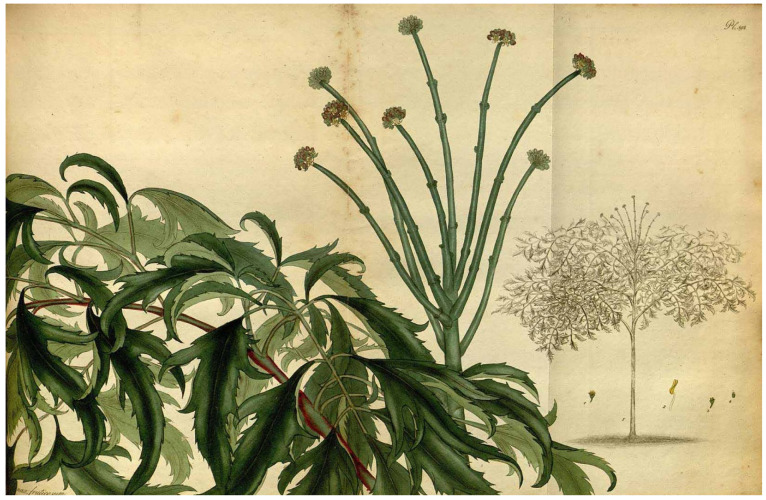
Botanical illustration of *Polyscias fruticosa* (L.) Harms (as *Panax fruticosus* L.), family Araliaceae, from *The Botanist’s Repository*, Vol. 9 (1809), Plate 595, drawn by H.C. Andrews. The image depicts the compound, serrated leaves, thick inflorescence-bearing stems, and the overall growth habit of the plant. Source: Missouri Botanical Garden, St. Louis, MO, USA. Public domain. Available online at http://www.plantillustrations.org/illustration.php?id_illustration=111562 (accessed on 30 April 2025) [28].

Leaves are alternate, bipinnate to tripinnate and imparipinnate, with long petioles (7–16 cm) and total dimensions reaching up to 29 × 25 cm. Each leaf comprises 7 to 15 pinnae, with pinnules bearing two–three pairs per pinna. The leaflets are narrowly lanceolate to elliptical (0.3–3 cm wide, 4.5–5 cm long), cuneate at the base, long-acuminate at the apex, and irregularly dentate to laciniate along the margins. Leaf coloration ranges from deep green in shade-grown specimens to yellowish hues or variegated forms in sun-exposed or horticultural varieties (Figure 1) [28]. The intricate leaf morphology and variable coloring enhance its visual distinctiveness and ornamental appeal [1,2].

**Figure 2:**
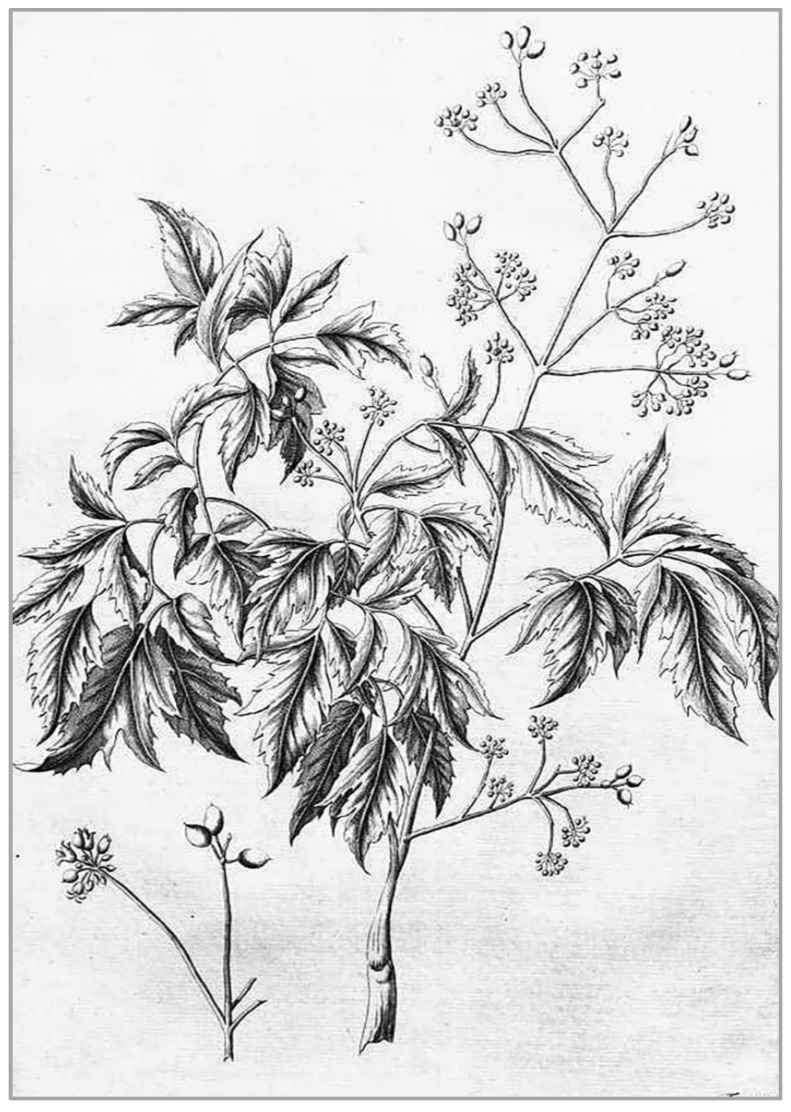
*Polyscias fruticosa* (L.) Harms (synonym: *Panax fruticosus* L.). Historical botanical illustration by Georgius Everhardus Rumphius (Rumpf), from *Herbarium Amboinense*, Vol. 4, Tab. XXXIII, p. 78 (1743). Image obtained from the website of the Missouri Botanical Garden, St. Louis, MO, USA. Public domain. Available online at http://www.plantillustrations.org/illustration.php?id_illustration=121516 (accessed on 13 June 2025) [29].

The inflorescences are terminal or axillary panicles (10–30 cm) composed of numerous umbellate cymes (12–20 per inflorescence). Flowers are actinomorphic, hermaphroditic, or functionally male, with articulated pedicels, a ring-like gamosepalous calyx, five deciduous ovate petals (2–4 mm), and five stamens with beige filaments and ovate yellow to white anthers. The inferior ovary comprises two–three carpels with initially fused styles that may separate or twist during fruit maturation (Figure 2) [29]. The fruit is a laterally compressed drupe (3–4 mm in diameter), dark purple to black at maturity, containing two flattened pyrenes and a seed with smooth endosperm [1,2].

The species belongs to the genus *Polyscias*, originally described by J.R. and G. Forster in 1775. Historically heterogeneous, the genus was redefined by Lowry and Plunkett [3], who integrated six previously separate genera (*Arthrophyllum*, *Gastonia*, *Reynoldsia*, *Cuphocarpus*, *Tetraplasandra*, and *Munroidendron*) based on molecular phylogenetic data. As a result, the recircumscribed genus now includes approximately 159 accepted species, systematically organized into ten subgenera. *P. fruticosa* is classified within *Polyscias* sensu stricto, a lineage characterized by articulated pedicels, sheathing petiole bases, and a distinct foliar fragrance upon drying [3].

The native range of *P. fruticosa* includes Southeast Asia and western Oceania, from where it has spread through human-mediated cultivation. It is now distributed in Vietnam, Laos, Cambodia, the Philippines, Indonesia, Papua New Guinea, India, China, and many Pacific and Caribbean islands, including Cuba, Puerto Rico, and the Virgin Islands [2]. In Cuba, the plant has been cultivated since at least the late 19th century and is commonly used in urban landscaping as a hedge or potted ornamental. It prefers well-drained, fertile soils and thrives under full sun or partial shade, although its growth is generally slow and benefits from periodic pruning and organic amendments.

Genetic and morphological studies conducted in Vietnam revealed high phenotypic plasticity and significant intraspecific variation within *P. fruticosa*. Using leaf morphology and internal transcribed spacer (ITS) rDNA sequencing, over 20 morphotypes were differentiated among 23 accessions, including several subspecies and potential hybrids. These findings support the hypothesis of regional domestication and extensive cultivar development driven by traditional medicinal use and esthetic selection [30].

According to Plants of the World Online (POWO) [31], *P. fruticosa* has 18 accepted synonyms, reflecting historical taxonomic revisions. Notable examples include *Panax fruticosus* L., *Nothopanax fruticosus* (L.) Miq., *Aralia fruticosa* (L.) L.H. Bailey, and *Tieghemopanax fruticosus* (L.) R.Vig. The name *Polyscias* is derived from the Greek “poly” (many) and “skia” (shade), referring to the plant’s dense, spreading foliage [2].

From an ethnopharmacological standpoint, *P. fruticosa* is a widely used medicinal species. In Vietnam, it is traditionally known as “đinh lăng” and used as a tonic, adaptogen, and treatment for inflammation, respiratory disorders, and fatigue [9]. In Indonesian jamu medicine, it features in postpartum and gynecological formulations. Ethnobotanical records from the Philippines and Oceania describe its use as an antipyretic, analgesic, and diuretic, applied as decoctions or topical poultices [9]. The widespread use of *P. fruticosa* in traditional medicine aligns with its confirmed phytochemical richness, including oleanane-type triterpenoid saponins (e.g., polysciosides A–K), chikusetsusaponin IVa, flavonoids, polyacetylenes, and sterols [3,4,5,6,7,8,9,10,11]. Pharmacological studies have confirmed its antioxidant, anti-inflammatory, antidiabetic, immunomodulatory, and antimicrobial effects [12,13,14,15,16,17].

Overall, *P. fruticosa* represents a phytochemically and ethnomedically important species. Its morphological plasticity, wide geographic distribution, and medicinal versatility make it a promising subject for integrative research spanning taxonomy, molecular phylogenetics, and evidence-based phytotherapy.

## 4. Micropropagation Strategies for *Polyscias fruticosa*

### 4.1. Historical Background of In Vitro Studies on Polyscias fruticosa

The development of in vitro culture systems for *Polyscias fruticosa* has played a critical role in advancing plant biotechnology, particularly with respect to secondary metabolite production in the Araliaceae family. Foundational studies were conducted at the K.A. Timiryazev Institute of Plant Physiology (IPPRAS, Moscow, Russia) in the mid-to-late 20th century, laying the groundwork for successful cultivation of this species under controlled conditions. Led by Professor Raisa G. Butenko, the IPPRAS team pioneered the induction of stable cell lines in medicinal plants, including *Panax ginseng*, *Catharanthus roseus*, and *Dioscorea deltoidea*, providing both methodological and conceptual frameworks for subsequent research on lesser-known taxa such as *P. fruticosa* [32].

The first callus and suspension cultures of *P. fruticosa* were initiated using leaf explants from greenhouse-grown donor plants, stabilized aseptically, and incorporated into the All-Russian Collection of Plant Cell Cultures—one of the largest repositories of medicinal plant cell lines in Eastern Europe. Suspension cultures demonstrated stable biosynthesis of triterpenoid saponins, including polysciosides and their derivatives, with concentrations ranging from 0.5 to 3.0% dry weight. This capacity supports the pharmacological potential of *P. fruticosa* as an adaptogen and immunomodulator and reinforces its viability as a phytopharmaceutical alternative to *Panax* species [32].

The scale-up of cultures was achieved in 20 L and 75 L bubble-type bioreactors operated in semi-continuous regimes, ensuring reproducible biomass accumulation and maintaining metabolite productivity. Notably, two compounds—*Polyscias fruticosa* saponin (PFS) and ladyginoside A—were identified and quantified at concentrations of 0.91 mg·g^−1^ DW and 0.77 mg·g^−1^ DW, respectively, surpassing yields obtained in flask cultures [17]. The resulting biomass was utilized for phytopharmaceutical development, including the commercial adaptogen Vitagmal©, produced by SMC ‘Biopharmtox’, St. Petersburg, Russia, and introduced in Russia in the 1990s—one of the earliest examples of a semi-industrial product based on plant cell cultures [32,33].

To preserve biosynthetic competence and genetic stability, selected cell lines were cryopreserved in liquid nitrogen at −196 °C. Post-thaw recovery confirmed the retention of both metabolic competence and regenerative capacity, validating the IPPRAS cryobank as an effective model for preserving elite *P. fruticosa* genotypes [33].

Currently, *P. fruticosa* is one of the few Araliaceae species with comprehensively documented in vitro protocols, including callogenesis, suspension culture, bioreactor cultivation, and cryopreservation. These developments provide a robust platform for further studies in micropropagation, metabolite enhancement, and sustainable biomass production [17,33].

### 4.2. Biotechnological Strategies for In Vitro Culture of Polyscias fruticosa

In vitro culture techniques are fundamental tools in the biotechnological propagation, conservation, and phytochemical exploitation of medicinal plants. They enable year-round controlled biomass production, independent of environmental fluctuations, and are widely used in pharmaceutical and cosmetic applications [34]. Tissue culture enables the rapid, clonal propagation of selected genotypes and allows for the stabilization and enhancement of secondary metabolite production through elicitation and genetic transformation strategies [35]. Such methodologies are particularly relevant for slow-growing species, those with limited natural distribution, or taxa threatened by overharvesting, offering a sustainable and scalable alternative to conventional cultivation practices [34,36].

*Polyscias fruticosa*, a pharmacologically promising member of the Araliaceae family, has gained attention due to its rich content of bioactive secondary metabolites, particularly triterpenoid saponins. However, the traditional cultivation of this species is constrained by low biomass yield, poor seed germination rates, and an extended vegetative period, significantly limiting its applicability in industrial phytopharmaceutical production [17,22].

In vitro propagation systems represent a viable solution to these constraints, providing rapid, reproducible, and environmentally independent means of generating uniform plant material. These systems also facilitate metabolic studies and the targeted enhancement of pharmacologically relevant compounds through the use of biotic and abiotic elicitors. The present study reviews and compares the efficacy of the main regenerative strategies for *P. fruticose*—namely, somatic embryogenesis, adventitious root culture, and suspension culture—with particular emphasis on their applicability in phytochemical and pharmacognostic research.

#### 4.2.1. In Vitro Regeneration of *Polyscias fruticosa*: Embryogenesis and Shoot Apices

Somatic embryogenesis remains a widely adopted method for clonal propagation in plant biotechnology [37,38]. Within the Araliaceae family, this technique has been successfully applied to species such as *Panax ginseng*, *Eleutherococcus senticosus*, and *Aralia elata* [35,36,37,38]. The process involves the differentiation of somatic cells into embryogenic structures that mimic the developmental stages of zygotic embryos—globular, heart-shaped, torpedo, and cotyledonary. The embryogenic transition can proceed directly from explants or indirectly through a callus phase, offering high regeneration efficiency and facilitating adaptation to bioreactor-based systems [39,40].

Meristem-based micropropagation utilizes shoot apices or axillary buds as explants and exploits the high mitotic activity and low differentiation status of meristematic tissues [41]. This approach allows for the rapid proliferation of genetically stable and pathogen-free plantlets, minimizing somaclonal variation and chimerism. It is particularly advantageous when maintaining clonal fidelity is essential for phytopharmaceutical applications.

Both somatic embryogenesis and meristematic propagation have proven effective for *P. fruticosa*, enabling the generation of uniform, high-quality explants for downstream metabolic and biotechnological applications [42].

Tram et al. [43] established an efficient protocol for somatic embryogenesis of *P. fruticosa* using leaf explants from two-year-old ex vitro plants. Callus induction was achieved on MS [44] medium with 0.3 mg·L^−1^ 2,4-D, 2.0 mg·L^−1^ BAP, and 7 mL/L coconut water. Subculturing onto MS medium with 0.5 mg·L^−1^ BAP and 100 mL/L CW resulted in a high embryogenesis rate (75.5%). Embryos developed into shoots on MS medium with 0.1 mg·L^−1^ kinetin and 150 mL/L CW, producing on average 6.3 shoots per explant.

These results indicate that the combination of BAP and CW is highly effective in promoting embryogenesis and shoot conversion, confirming the morphogenic potential of coconut-derived additives.

Phuong et al. [24] established embryogenic suspension cultures from leaf-derived calli in liquid MS medium with 2,4-D or NAA (0.5–2.0 mg·L^−1^), achieving the highest biomass (5.7 g/flask) and somatic embryo yield (489/flask) at 1.5 mg·L^−1^ NAA. The occurrence of rhizogenic cells suggests a robust regeneration pathway combining embryogenesis and rooting.

Sakr et al. [23] developed a shoot-apex-based protocol for juvenile plants using B5 [45] medium enriched with BAP and kinetin, reaching up to 6.7 shoots per explant. Rooting on MS medium with 1.0 mg·L^−1^ NAA produced 6.42 cm long roots, with over 80% acclimatization success.

These protocols highlight the species’ high regenerative plasticity and suggest that adjusting cytokinin/auxin ratios is key to optimizing micropropagation. This regenerative responsiveness underscores the biotechnological potential of *P. fruticosa*, enabling its application in efficient biomass propagation, metabolic profiling, and long-term conservation strategies.

A simplified micropropagation protocol was introduced by Pandya et al. [22], using MS medium supplemented only with kinetin (0.5 mg·L^−1^). Shoot emergence occurred within 20 d, and the complete regeneration cycle—including elongation, rooting, and hardening—was achieved in 60 d. This method, by eliminating auxins and reducing hormonal inputs, shortened the production time compared to conventional 80 d systems and offers a cost-effective alternative for large-scale propagation. Its minimalistic design highlights the intrinsic morphogenetic potential of *P. fruticosa*, even under reduced hormonal stimulation.

Collectively, somatic embryogenesis and meristem-based propagation strategies offer robust platforms for the rapid and standardized production of *P. fruticosa* plantlets. These plantlets can be used for callus induction, genetic transformation, elicitor studies, and secondary metabolite production. Meanwhile, meristematic approaches remain critical for maintaining genetic fidelity, eliminating pathogens, and ensuring consistency in phytopharmaceutical applications.

In the authors’ view, these complementary strategies fulfill both research and industrial objectives: while embryogenic systems are suited for scalable production and bioreactor implementation, meristem culture offers quality assurance for pharmaceutical-grade propagation. The integration of both approaches is essential for ensuring efficient, reproducible, and sustainable exploitation of *P. fruticosa* in modern biotechnology.

#### 4.2.2. Adventitious Root Cultures of *Polyscias fruticosa*: Induction, Optimization, and Saponin Production

The induction of callus and regeneration of adventitious roots constitute essential in vitro techniques for obtaining root-derived organs capable of synthesizing secondary metabolites of pharmacological relevance, especially when full-plant regeneration is not necessary [46,47,48,49]. Callus, as an undifferentiated tissue, can be hormonally directed towards root morphogenesis using specific auxins, such as 2,4-dichlorophenoxyacetic acid (2,4-D), indole-3-acetic acid (IAA), indole-3-butyric acid (IBA), or α-naphthaleneacetic acid (NAA), either individually or in combination with cytokinins [50,51,52].

Root cultures offer the opportunity to apply elicitation strategies that enhance secondary metabolite biosynthesis via biochemically induced stress responses. Elicitors such as jasmonic acid (JA), yeast extract (YE), or mannitol activate cellular defense mechanisms and stimulate the accumulation of bioactive compounds, providing a promising approach for the optimized production of high-value phytochemicals [50,53,54,55].

In the case of *P. fruticosa*, this approach is particularly relevant, as roots are the primary site of biosynthesis and storage of triterpenoid saponins a class of compounds known for their anti-inflammatory, cytotoxic, and antihyperglycemic properties [56,57]. However, their natural concentration is relatively low, and the plant’s cultivation cycle spans three to five years, rendering conventional harvesting economically inefficient [22,58,59].

In the study by Lộc et al. [60], adventitious roots were induced from leaf explants of three-year-old greenhouse-grown *P. fruticosa* plants. The explants were cultured on MS medium supplemented with 2,4-D at concentrations ranging from 0 to 4.0 mg·L^−1^. The highest callus induction rate (100%) was observed at 2.0 mg·L^−1^ 2,4-D. After four weeks, the resulting calli were transferred to MS medium containing 1.0 mg·L^−1^ IBA and 0.1 mg·L^−1^ NAA, which promoted robust root formation. The induction and development of roots followed a two-step protocol involving auxin-specific stimulation for morphogenic differentiation. Qualitative analysis confirmed the presence of saponins in the in vitro-derived roots, with a dry weight content of 1.67%, corresponding to 83.5% of the concentration found in field-grown roots (1.99%). The identification of saponins was conducted using phytochemical tests and gravimetric analysis.

Subsequently, Le et al. [12] evaluated the effects of three elicitors—JA, YE, and mannitol—on secondary metabolite production in *P. fruticosa* adventitious root cultures. Roots obtained through NAA-induced rhizogenesis (2.0 mg·L^−1^) were transferred to media supplemented with varying concentrations of elicitors: JA (0.5, 1.0, and 2.5 mM), YE (100, 300, and 500 mg·L^−1^), and mannitol (50, 100, and 150 mM). The highest total saponin content (167.19 ± 3.29 mg·L^−1^ extract, expressed as aescin equivalents) was recorded in cultures treated with 2.5 mM JA, representing more than a threefold increase compared to the control (54.08 mg·L^−1^) and surpassing the levels found in wild-type roots (137.37 mg·L^−1^). Saponins were extracted using ethanol-based solvent systems and quantified spectrophotometrically via vanillin–sulfuric acid assay.

These results demonstrate that elicitation of *P. fruticosa* root cultures significantly enhances the biosynthesis of triterpenoid saponins, offering an efficient and reproducible system for the scaled production of pharmacologically valuable compounds.

This approach ensures metabolic stability of the root system in vitro, reduces production time, and may serve as an ecologically and economically viable alternative to field cultivation.

#### 4.2.3. Cell Suspension Cultures of *Polyscias fruticosa*: Optimization, Bioreactor Scaling, and Production of Bioactive Triterpenoids

Plant cell suspension cultures offer a controlled and scalable system for the production of high-value secondary metabolites. Typically initiated from friable callus derived from explants, these cultures are maintained in agitated liquid MS-based media under regulated temperature and photoperiod conditions. Agitation and aeration ensure uniform cell proliferation and support metabolite biosynthesis, independent of environmental fluctuations [61]. This approach has been validated in numerous medicinal plants, including *Taxus* spp., *Panax* spp., and *Catharanthus roseus*, for the production of taxanes, ginsenosides, and alkaloids, respectively [62,63]. Suspension cultures provide high reproducibility and process standardization, while allowing the synthesis of both native and novel compounds via metabolic reprogramming [63].

Nevertheless, these systems face certain limitations, such as genetic instability, low metabolite yields, and microbial contamination. Optimization strategies—such as the selection of productive cell lines, modulation of plant growth regulators, elicitor treatments, and bioreactor scaling—have been employed to overcome these challenges. Within the Araliaceae family, *Polyscias fruticosa* has emerged as a promising candidate for cell suspension culture due to its ability to biosynthesize bioactive triterpenoid saponins [62,63].

In an early study, Kim et al. [25] established a suspension culture from 30 d-old stem-derived callus. Cultures were maintained in MS medium with 30 g·L^−1^ sucrose, 1.0 mg·L^−1^ BAP, and 0.5 mg·L^−1^ 2,4-D under a 12 h/12 h light/dark cycle at 25 ± 2 °C and 120 rpm. After 16 days, dry biomass reached 0.43 g per flask (a 3.7-fold increase), and oleanolic acid accumulated to 25.4 mg·L^−1^, nearly five times higher than in in vitro leaves (5.2 mg/g DW) [25]. Further experiments by the same group [26] revealed that sucrose (30 g·L^−1^) outperformed other carbon sources in biomass promotion, while elicitors like yeast extract and silver nitrate induced a shift towards secondary metabolism at the expense of biomass. This suggests that a trade-off exists between growth and metabolite accumulation, which must be optimized depending on the intended application. In comparative studies, the application of malonyl ginsenosides (M-GS) as elicitors in two cell lines—6a (*P. fruticosa*) and VDK (*P. filicifolia*)—revealed contrasting responses [27]. The 6a line showed a 79.7% increase in polyscioside E and a 70.7% rise in ladyginoside A, despite a ~45% reduction in biomass. In contrast, the VDK line exhibited lower biomass, but a 63.3% increase in only polyscioside A, highlighting cell-line-specific responses.

Scalability was demonstrated by Titova et al. [17], who used a 20 L bubble-type bioreactor operated semi-continuously. Peak biomass reached 6.8 g·L^−1^ DW, with productivity of 0.32 g·L^−1^·d^−1^and over 70% cell viability. Saponin content significantly increased: PFS (3-O-[β-D-glucopyranosyl-(1→4)-β-D-glucuronopyranosyl] oleanolic acid 28-O-β-D-glucopyranosyl ester) reached 0.91 mg·L^−1^ DW (82% higher than in flask cultures), while ladyginoside A reached 0.77 mg·L^−1^ DW (3.5-fold increase). The extracts exhibited antimicrobial activity (MIC = 250–2000 µg·mL^−1^) against *Staphylococcus aureus*, MRSA, *Pseudomonas aeruginosa*, and *Escherichia coli*, outperforming leaf-derived extracts (MIC = 4000 µg·mL^−1^). Antioxidant activity was also confirmed via DPPH and TEAC assays, indicating the retention of pharmacologically active compounds under in vitro conditions [17].

The suspension culture system of *P. fruticosa* provides a biotechnologically robust platform for triterpenoid saponin production, with documented scalability and responsiveness to elicitation. The observed trade-off between growth and metabolite synthesis, alongside cell-line-specific metabolic behavior, underlines the need for tailored optimization. Bioreactor systems, when properly configured, enhance both productivity and phytochemical enrichment, positioning *P. fruticosa* as a viable candidate for pharmaceutical-scale bioproduction.

### 4.3. In Vitro Propagation and Phytoremediation Potential of Polyscias fruticosa

Beyond its pharmacological significance, *P. fruticosa* exhibits notable potential for phytoremediation, particularly in the detoxification of heavy metals from contaminated environments. A comparative study by Pandya et al. [64] demonstrated that plants regenerated through in vitro methods accumulate significantly higher levels of lead (Pb) and cadmium (Cd) than conventionally propagated (in vivo) specimens.

In this study, in vitro-derived plants were obtained via a shoot apex micropropagation protocol using apical meristems cultured on Murashige and Skoog (MS) medium supplemented solely with 0.5 mg·L^−1^ kinetin, without the addition of auxins. This simplified, hormone-restricted protocol enabled the generation of genetically uniform and physiologically vigorous plantlets. Following in vitro rooting and successful acclimatization, the plants were transplanted to Pb- and Cd-contaminated soils to assess phytoremediation capacity.

At the highest tested soil concentration of Pb (800 mg·kg^−1^ ), in vitro-propagated plants accumulated 1420 mg·kg^−1^ in roots and 979 in leaves, compared to 1331 and 667, respectively, in in vivo-grown controls. Similarly, for Cd, in vitro-derived plants exhibited greater accumulation (89.09 in roots and 33.33 in leaves) than in vivo plants (80.81 and 23.13, respectively).

These results suggest that the superior phytoremediation performance of in vitro-regenerated plants may be due to their increased uniformity, higher metabolic rates, and greater physiological adaptability under stress conditions. Moreover, tissue-culture-derived lines may be selectively enriched or genetically engineered to further improve metal tolerance and detoxification efficiency.

Given the global urgency of remediating heavy-metal-polluted soils and the scalable nature of micropropagation systems, *P. fruticosa* represents a viable candidate for eco-friendly biotechnological applications. Its potential applicability in post-industrial soil recovery, urban ecological restoration, and as part of phytoprotective green infrastructure highlights the need for further investigation.

### 4.4. Author’s Perspective and Future Directions

The accumulated data confirm that *Polyscias fruticosa* exhibits remarkable morphogenetic plasticity across diverse in vitro systems, including somatic embryogenesis, shoot apex-based micropropagation, adventitious root cultures, and cell suspension cultures. This developmental versatility enables both rapid clonal propagation and targeted biosynthesis of pharmacologically active secondary metabolites, particularly triterpenoid saponins [12,17,22,24,25,26,27].

Among these strategies, somatic embryogenesis and meristem-based protocols ensure the highest genetic stability, making them ideal for transformation, virus elimination, and germplasm conservation. In contrast, adventitious root and suspension cultures offer rapidly proliferating undifferentiated biomass suitable for elicitor-driven secondary metabolism and phytopharmaceutical production [17,23,24,25].

Comparative elicitation studies highlight the complementary potential of these systems. Adventitious root cultures treated with 2.5 mM jasmonic acid (JA) achieved the highest saponin content (167.19 mg·L^−1^), exceeding that of both untreated and wild-type roots. Meanwhile, suspension cultures maintained in 20 L bioreactors demonstrated superior biomass accumulation (6.8 g·L^−1^ DW) and elevated yields of individual saponins, such as PFS (0.91 mg·g^−1^ DW) and ladyginoside A (0.77 mg·g^−1^ DW), outperforming conventional flask cultures [12,17]. These findings suggest that adventitious roots respond more strongly to elicitation, while suspension cultures are better suited for upscaling and process optimization under bioreactor conditions.

Despite these promising outcomes, key knowledge gaps remain. The molecular mechanisms underlying saponin biosynthesis, hormonal signaling, and elicitor-induced responses in *P. fruticosa* have yet to be elucidated. To date, no transcriptomic, metabolomic, or proteomic analyses have been conducted for any in vitro culture type of this species. Furthermore, there is a lack of direct comparative analyses of metabolite profiles between adventitious roots and suspension cultures using harmonized extraction procedures and chromatographic parameters. Additionally, the long-term genetic and metabolic stability of in vitro-derived lines, particularly under elicitor treatment or bioreactor conditions, requires further assessment.

Future research should prioritize the following directions:

- The integration of transcriptomic, metabolomic, and proteomic analyses to unravel biosynthetic regulatory networks;
- The optimization of culture parameters using metabolic modeling, combinatorial elicitation, and environmental stimuli (e.g., light, temperature);
- Comparative profiling of triterpenoid production across culture types and elicitation regimes;
- The evaluation of genetic and biochemical stability under long-term and large-scale in vitro propagation.

For industrial translation, both adventitious root and suspension cultures should be scaled up in air-lift or stirred-tank bioreactors, coupled with life-cycle assessment (LCA) and techno-economic analysis. This would facilitate the standardized, sustainable production of targeted saponins with validated pharmacological properties, potentially reducing dependence on wild or field-grown biomass.

From an applied perspective, *P. fruticosa* also emerges as a promising candidate for phytoremediation. In vitro-regenerated plants demonstrated enhanced accumulation of heavy metals such as lead and cadmium, underscoring their dual function as both phytopharmaceutical and environmental bioresources [65]. Ultimately, integrating micropropagation with metabolic engineering and stress conditioning may unlock the full biotechnological value of this underutilized Araliaceae species.

## 5. Chemical Composition and Structural Diversity of Bioactive Compounds in *Polyscias fruticosa*

### 5.1. Triterpenoid Saponins

Triterpenoid saponins are a class of glycosidic secondary metabolites derived from pentacyclic triterpenes such as oleanolic acid and are widely distributed across the Araliaceae family. These compounds exhibit a broad spectrum of pharmacological activities, including anti-inflammatory, antidiabetic, immunomodulatory, cytotoxic, and neuroprotective effects, which are largely attributed to their amphipathic structures that enable interactions with cellular membranes, enzymes, and signaling pathways [65,66,67,68]. Their relevance in drug discovery has gained particular attention due to their potential application as natural therapeutics or adjuvants in the treatment of metabolic, inflammatory, and oncological disorders [65]. In *P. fruticosa*, triterpenoid saponins constitute the dominant class of bioactive constituents, and their structural diversity provides a valuable basis for both pharmacological evaluation and biotechnological exploitation. Importantly, selected saponins have demonstrated immunostimulatory or antimicrobial properties, reinforcing their therapeutic value.

#### 5.1.1. Contextual Account of Triterpenoid Saponin Discovery in *Polyscias fruticosa*

The presence of triterpenoid saponins in *P. fruticosa* was first reported in the mid-1990s, marking a key milestone in the phytochemical exploration of this species (Table 1). In 1995, Chaboud and colleagues isolated a saponin from the leaves of *P. fruticosa*, identified as 3-O-[β-D-galactopyranosyl-(1→2)-β-D-glucuronopyranosyl] oleanolic acid [69]. This compound was characterized as a monodesmosidic saponin, featuring a disaccharide chain at the C-3 position of the oleanolic acid aglycone and lacking substitution at the C-28 position. Its structure confirmed the occurrence of oleanane-type saponins, which are typical for members of the Araliaceae family. The predominance of oleanane-type structures among the saponins isolated from *P. fruticosa* confirms its chemotaxonomic affiliation with other medicinally relevant Araliaceae species such as *Panax* and *Aralia*. The presence of shared compounds (e.g., chikusetsusaponin IVa) further supports this classification.

In 1996, Proliac et al. [5] reported the isolation of another oleanane-type saponin from the yellow-leaved variant of *P. fruticosa*, structurally identified as 3-O-[α-L-rhamnopyranosyl-(1→4)-β-D-glucuronopyranosyl]-28-O-β-D-glucopyranosyl oleanolic acid. Unlike the compound described by Chaboud et al. [69], this saponin exhibited a bisdesmosidic structure, with glycosidic substitutions at both the C-3 and C-28 positions of the aglycone, a feature commonly observed in Araliaceae saponins.

A major advancement occurred in 1998, when Vo Duy Huan and colleagues conducted a comprehensive phytochemical analysis of *P. fruticosa* leaf and root extracts, resulting in the isolation of eleven triterpenoid saponins [3]. Eight of these were novel compounds, designated polysciosides A–H. All were based on oleanolic acid aglycones and exhibited bisdesmosidic structures with varying sugar compositions and glycosidic linkages. Several of these novel saponins were subsequently reported to exhibit cytotoxicity against tumor cell lines or antioxidant potential. The structural variation among polysciosides A–H, particularly the nature and position of sugar moieties, is likely to influence membrane interaction and bioactivity, although systematic structure–activity relationship (SAR) studies are still lacking.

The remaining three compounds had previously been reported: ladyginoside A from *Ladyginia bucharica* [70], zingibroside R1 from *Panax zingiberensis* [3], and a compound later referred to as PFS (*Polyscias fruticosa* saponin), originally isolated from *Swartzia simplex* and identified as 3-O-[β-D-glucopyranosyl-(1→4)-β-D-glucuronopyranosyl] oleanolic acid 28-O-β-D-glucopyranosyl ester [71].

In 2003, Divakar and co-workers [6] isolated two additional novel saponins: oleanolic acid-28-O-β-D-glucopyranosyl-D-glucopyranosyl-rhamnopyranoside and 3-O-α-L-arabinopyranosyl-oleanolic acid-28-O-β-D-glucopyranosyl-D-glucopyranosyl-rhamnopyranoside. Although the linkages between sugar moieties were not fully elucidated, both compounds demonstrated significant immunostimulatory activity. They enhanced the phagocytic activity of leukocytes against *Candida albicans* in vitro and stimulated carbon clearance in mice. Importantly, neither compound exhibited toxicity in brine shrimp lethality assays (LC_50_ > 800 µg·mL^−1^) or in acute oral toxicity tests in Swiss albino mice at doses up to 1.5 g kg^−1^ body weight. These findings suggest that the immunomodulatory effects of certain saponins from *P. fruticosa* may be mediated through enhanced phagocytic responses and macrophage activation, although mechanistic studies at the molecular level remain to be conducted.

Since 2010, research has focused on expanding the inventory of triterpenoid saponins in *P. fruticosa*, aided by advances in chromatographic and spectroscopic techniques such as high-performance liquid chromatography (HPLC), gel filtration, high-resolution mass spectrometry (HR-MS), and multidimensional NMR spectroscopy. These modern approaches have improved structure elucidation and supported pharmacokinetic evaluations. However, no pharmacokinetic or bioavailability studies have yet been reported for individual *P. fruticosa* saponins in vivo, which limits their translational potential.

In 2016, Tran et al. [14] isolated three bisdesmosidic saponins from *P. fruticosa* leaves, including a novel compound designated as polyscioside I, which displayed the characteristic Araliaceae glycosylation pattern at both the C-3 and C-28 positions.

Further discoveries by Do et al. in 2020 [7] led to the isolation of four saponins from the leaves, two of which were newly identified and named polyscioside J and polyscioside K. Notably, this study also confirmed—for the first time—the presence of chikusetsusaponin IVa in *P. fruticosa*, a compound previously reported in *Panax ginseng*, thereby reinforcing the chemotaxonomic relationship between *P. fruticosa* and other medicinally important genera of the Araliaceae family [8].

To date, at least 19 triterpenoid saponins have been isolated and structurally characterized from *P. fruticosa*. This group includes both unique compounds and saponins shared with other Araliaceae species, providing a robust foundation for future pharmacological and biotechnological investigations. However, the biosynthetic pathways responsible for this diversity remain poorly characterized, and the correlation between specific structures and bioactivities has yet to be systematically explored. Further studies integrating metabolomics and enzymatic profiling are warranted to clarify these relationships.

Despite the growing inventory of saponins in *P. fruticosa*, their pharmacological relevance remains underexplored in preclinical models. Most reports are limited to in vitro assays, and few compounds have been tested for toxicity, bioavailability, or efficacy in animal systems. A systematic evaluation of their immunomodulatory and cytotoxic properties, particularly for bisdesmosidic saponins, could identify promising therapeutic leads. Furthermore, understanding how glycosylation patterns influence biological activity may guide future efforts in semi-synthetic modification and metabolic engineering.

#### 5.1.2. Extraction Strategies for Triterpenoid Saponins

Given the therapeutic relevance of triterpenoid saponins, considerable efforts have been made to develop efficient extraction methods for *P. fruticosa* plant material. Aqueous ethanol-based protocols remain standard, but recent innovations offer greater selectivity and sustainability.

Among these, ultrasound-assisted extraction (UAE) has demonstrated notable efficacy. Khoang et al. [58] applied UAE in combination with response surface methodology (RSM) to optimize the recovery of saponins from *P. fruticosa* roots. Under optimal conditions, i.e., 60 °C, 60% ethanol, 65 min, and 180 W ultrasonic power, the total saponin yield reached 14.5% of the dry root mass, and the extract contained approximately 41 mg·g^−1^ of saponins. The extract showed cytotoxicity against A549, HepG2, PC-3, and HeLa cell lines.

In a complementary study, Chuyen et al. [72] evaluated the influence of extraction parameters on both saponin recovery and antioxidant activity. Extraction with 40% ethanol at 50 °C for 90 min, using a solvent-to-solid ratio of 40:1, resulted in high yields of saponins and co-extracted phenolic compounds. The obtained extracts demonstrated enhanced antioxidant capacity, suggesting saponin–polyphenol synergy.

Such optimization supports both pharmacological efficacy and product standardization.

#### 5.1.3. Pharmacological Potential of *Polyscias fruticosa* Saponins

Numerous pharmacological studies have validated the traditional uses of *P. fruticosa* and demonstrated the broad spectrum of biological activities exerted by its triterpenoid saponins [4,6,7,14,16,17]. One of the most extensively studied compounds is PFS, identified as 3-O-[β-D-glucopyranosyl-(1→4)-β-D-glucuronopyranosyl] oleanolic acid 28-O-β-D-glucopyranosyl ester (**13**). This saponin exhibited significant inhibitory activity against key carbohydrate-hydrolyzing enzymes: porcine pancreatic α-amylase (IC_50_ = 27.1 µg·mL^−1^, ~21.2 µM) and yeast α-glucosidase (IC_50_ = 440.5 µg·mL^−1^, ~344 µM) [4,57]. In vivo, oral administration of PFS at a dose of 100 mg·kg^−1^ significantly reduced postprandial blood glucose levels in mice subjected to an oral sucrose tolerance test, resulting in decreases of 16.6% at 30 min and 27.9% at 60 min, along with a reduced area under the curve (AUC_0–120_ min) compared to controls [57]. These results confirm its potential in metabolic disorder management.

Another triterpenoid saponin identified in *P. fruticosa* is chikusetsusaponin IVa (**19**) [7], a compound with well-documented pharmacological activity. It possesses anti-inflammatory properties by suppressing the nuclear factor kappa B (NF-κB) signaling pathway and inhibiting activation of the NOD-like receptor protein 3 (NLRP3) inflammasome, leading to reduced expression of pro-inflammatory mediators such as TNF-α, IL-6, COX-2, and iNOS [73,74,75,76]. Under oxidative stress conditions, it enhances endogenous antioxidant defenses by upregulating superoxide dismutase (SOD), glutathione (GSH), and catalase (CAT), thereby mitigating cellular oxidative damage [74,75]. It also exerts significant antihyperglycemic effects by activating AMP-activated protein kinase (AMPK), which promotes glucose uptake and fatty acid oxidation via increased GLUT4 translocation and CPT-1 activation in insulin-resistant skeletal muscle cells [75,76]. In metabolic dysfunction models induced by high-fat diets, chikusetsusaponin IVa improves insulin sensitivity, regulates lipid profiles, and modulates macrophage polarization in adipose tissue [73,75].

Moreover, chikusetsusaponin IVa exhibits anticancer activity by inducing cell cycle arrest and apoptosis, suppressing metastasis-related pathways such as Wnt/β-catenin and IL-6/STAT3, and inhibiting matrix metalloproteinases (MMP-2 and MMP-9) [75]. It also demonstrates neuroprotective and cardioprotective effects through activation of SIRT1 and ERK1/2 pathways, reduction in ROS, and maintenance of mitochondrial function [75]. Its confirmed occurrence in *P. fruticosa* highlights its relevance and warrants further mechanistic research.

In addition to individual saponins, saponin-rich extracts from *P. fruticosa* have exhibited notable pharmacological effects. Despite this, polysciosides J (**17**) and K (**18**) alone showed low cytotoxicity, whereas saponin-enriched extracts were active against cancer cells, likely due to synergy with polyphenols [7]. These results support the potential therapeutic advantage of standardized phytocomplexes and encourage further in vivo and mechanistic studies.

Extracts derived from *P. fruticosa* suspension cell cultures grown in bubble-type bioreactors, containing triterpenoid saponins such as PFS and ladyginoside A (**11**), demonstrated potent antibacterial activity. In vitro assays revealed inhibitory effects against *Escherichia coli*, *Staphylococcus aureus*, and *Candida albicans*, with minimum inhibitory concentrations (MICs) of 250, 500, and 500 µg·mL^−1^, respectively [17]. The MIC for *E. coli* was markedly lower than that of extracts from greenhouse-grown leaves (MIC = 4000 µg·mL^−1^), suggesting enhanced antimicrobial potential of in vitro-derived biomass. The presence of saponins supports their contribution to antimicrobial activity.

Zingibroside R1 (**12**) is another confirmed oleanane-type saponin in *P. fruticosa*. In a *Caenorhabditis elegans* model, it demonstrated antioxidant and anti-aging properties by reducing intracellular ROS levels, enhancing locomotion, increasing resistance to oxidative and thermal stress, and extending lifespan [77]. In vitro, it inhibited glucose uptake in tumor cells and showed cytotoxicity against MT-4 human T-lymphocytes and Ehrlich ascites tumor cells (IC_50_ ≈ 91 µM) [78]. Antiviral activity against HIV-1 was also reported at subtoxic concentrations, with greater efficacy than glycyrrhizin [78]. In silico analyses using PASS software (version 2019) predicted high probabilities for antioxidant (Pa = 0.829), free radical scavenging (Pa = 0.411), and antihypoxic activity (Pa = 0.378), while PharmaExpert software was used to analyze possible synergistic or additive effects of these phytoconstituents [78]. Further predictions suggest neuroprotection via various mechanisms, including lipid peroxidation inhibition and NGF agonism [79]. These multifaceted effects justify further pharmaceutical investigation of Zingibroside R1.

From a pharmacotechnical perspective, the chemical stability of triterpenoid saponins is a crucial factor affecting extract quality. Le et al. [80] demonstrated that storage temperature significantly influences the integrity of ethanol extracts from *P. fruticosa* leaves. After 30 d, samples stored at 5 °C retained over 90% of their initial saponin content, whereas those stored at 30 °C and 60 °C exhibited reductions of 29.65 and 57.82%, respectively. These losses were accompanied by proportional declines in antioxidant activity, confirming that elevated storage temperatures accelerate saponin degradation and diminish biological efficacy. Therefore, cold storage is essential to maintain extract quality and efficacy.

#### 5.1.4. Proposed Biosynthetic Pathway of Triterpenoid Saponins (TSBP) in *Polyscias fruticosa*

**Figure 3:**
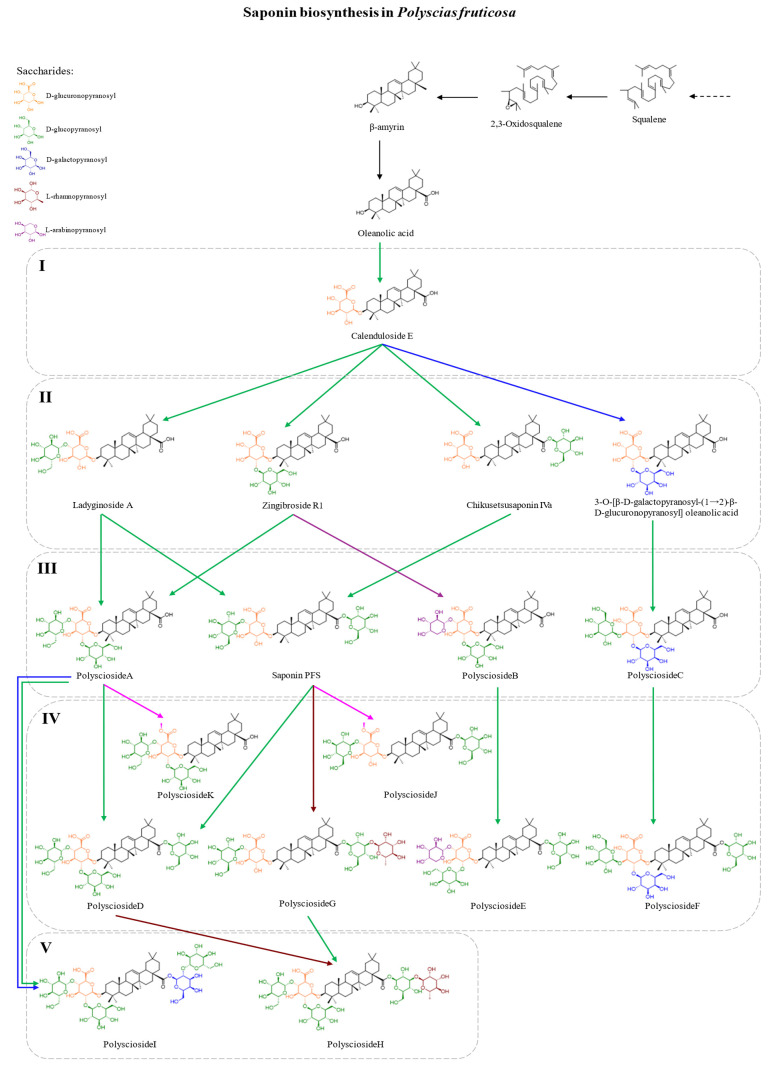
Proposed biosynthetic pathway of triterpenoid saponins in *Polyscias fruticosa*.

Based on the available literature and comparative analyses, a complete triterpenoid saponin biosynthetic pathway (TSBP) is proposed here for the first time in *P. fruticosa* (Figure 3). This reconstruction integrates structural and biochemical data from closely related Araliaceae species, such as *Aralia elata* [81], *Aralia spinosa* [82], and *Panax ginseng* [83].

In *P. fruticosa*, the TSBP proceeds via the canonical cytosolic mevalonate (MVA) pathway, yielding isopentenyl diphosphate (IPP) and dimethylallyl diphosphate (DMAPP) as key isoprenoid precursors. Their condensation forms farnesyl pyrophosphate (FPP), which dimerizes to squalene. Oxidation by squalene epoxidase generates 2,3-oxidosqualene, which is cyclized by β-amyrin synthase into β-amyrin, the oleanane-type triterpenoid core predominant in this species [84]. This reaction is catalyzed by oxidosqualene cyclases (OSCs), which determine the aglycone skeleton; in *P. fruticosa*, β-amyrin predominates, whereas dammarane- and ursane-type skeletons dominate in *Panax* and *Centella*, respectively [84].

Further oxidation at the C-28 position, likely catalyzed by cytochrome P450 monooxygenases from the CYP716 subfamily, converts β-amyrin to oleanolic acid (OA), the principal sapogenin in *P. fruticosa* [83]. This step mirrors CYP716A12 and CYP716A94 catalysis in *Medicago* and *Kalopanax*, and may involve CYP716A52v2-like or CYP716A75-like enzymes [83].

Subsequent glycosylation steps are catalyzed by UDP-dependent glycosyltransferases (UGTs), incorporating saccharides such as pentoses (L-arabinopyranosyl, Ara), deoxyhexoses (L-rhamnopyranosyl, Rha), hexoses (D-glucopyranosyl, Glc; D-galactopyranosyl, Gal), and uronic acids (D-glucuronopyranosyl, GluA). Sugar chains are typically attached at the C-3 hydroxyl and C-28 carboxyl groups. UGTs implicated in these reactions may include members of the UGT73, UGT74, and UGT94 subfamilies, as demonstrated in other Araliaceae. Initial glucosylation at C-3 is often followed by sugar branching at O-2, O-3, or O-4 of the glycosidic residues [83,84].

The proposed TSBP comprises five sequential stages of glycosylation and methylation, culminating in the structurally diverse array of saponins found in *P. fruticosa*. The first committed intermediate is presumed to be calenduloside E, formed by glycosylation of OA at C-3 with GluA. Although not yet isolated from *P. fruticosa*, this compound is likely a transient precursor rapidly converted to more complex saponins [85].

Glycosylation with Glc or Gal yields zingibroside R1 and ladyginoside A through substitutions at O-2 and O-4 of GluA, respectively. Alternatively, glycosylation at C-3 with Glc leads to chikusetsusaponin IVa, whereas conjugation of Gal to Calenduloside E produces 3-O-[β-D-galactopyranosyl-(1→2)-β-D-glucuronopyranosyl] oleanolic acid (Figure 3).

Additional sugar modifications diversify the saponin pool. For example, polyscioside A likely arises from Glc attachment to the sugar chains of zingibroside R1 or ladyginoside A. Polyscioside B is proposed to originate from zingibroside R1 via Ara substitution at O-4 of GluA, while polyscioside C may form through Glc addition at O-3 of the Gal–GluA chain. PFS (3-O-[β-D-glucopyranosyl-(1→4)-β-D-glucuronopyranosyl] oleanolic acid 28-O-β-D-glucopyranosyl ester) can be generated via esterification of C-28 in ladyginoside A or glycosylation of the same site in chikusetsusaponin IVa (Figure 3).

In addition to classical O-glycosylation, the TSBP in *P. fruticosa* appears to involve O-methylation of sugar moieties. At least six additional saponins arise from such modifications. Polyscioside D may result from Glc esterification of polyscioside A or from Glc attachment to the sugar chain of PFS. Polyscioside E is generated by Glc esterification at C-28 of polyscioside B, while polyscioside F forms analogously from polyscioside C. Polyscioside G likely results from Rha addition to the C-28 sugar chain of PFS. Furthermore, O-methylation of the GluA carboxyl in PFS or polyscioside A may yield polysciosides J and K, respectively.

Terminal steps in the pathway may involve the formation of polyscioside H and I. Polyscioside H could arise from Glc addition to polyscioside G or Rha conjugation to polyscioside D. Polyscioside I is proposed to form via the addition of a disaccharide unit (Gal–Glc) at the C-28 position of polyscioside A. A putative intermediate, 3-O-[β-D-glucopyranosyl-(1→2)-[β-D-glucopyranosyl-(1→4)]-β-D-glucuronopyranosyl] oleanolic acid 28-O-β-D-galactopyranosyl ester, is hypothesized to precede polyscioside I formation, although it has not yet been isolated from *P. fruticosa*.

Overall, the proposed TSBP in *P. fruticosa* suggests that its triterpenoid saponins are biosynthesized via both linear and convergent pathways. Some compounds, such as polyscioside E, are likely formed through a strictly linear progression (OA → calenduloside E → zingibroside R1 → polyscioside B → polyscioside E), whereas others, like polyscioside H, may derive from alternative branches. This model integrates structural, biochemical, and comparative insights, providing a robust framework for future experimental validation of saponin biosynthesis in *P. fruticosa* and supporting pathway engineering and synthetic biology applications. From the authors’ perspective, this model provides a biosynthetically coherent explanation of saponin diversity in *P. fruticosa*, integrating structural, enzymatic, and comparative data. It offers a foundation for future experimental validation and supports applications in metabolic engineering and synthetic biology.

#### 5.1.5. Concluding Remarks on Triterpenoid Saponins

Triterpenoid saponins constitute the principal class of bioactive metabolites in *P. fruticosa*, characterized by considerable structural diversity and a wide spectrum of pharmacological activities. To date, 19 saponins have been isolated and structurally characterized from this species, including both unique glycosides and compounds common to other Araliaceae taxa. Experimental studies have demonstrated their anti-inflammatory, antidiabetic, cytotoxic, antioxidant, and neuroprotective properties in various in vitro and in vivo models [8,14,16,17,20,56,57].

Efficient extraction techniques, particularly ultrasound-assisted extraction (UAE), have been developed to maximize saponin yield and preserve bioactivity. Moreover, the observed synergism between saponins and co-extracted phytochemicals enhances the therapeutic value of standardized saponin-rich extracts. Based on comparative phytochemical and biochemical data, a complete triterpenoid saponin biosynthetic pathway (TSBP) has been proposed for the first time in *P. fruticosa*, encompassing the mevalonate pathway, β-amyrin cyclization, oxidation to oleanolic acid, and subsequent glycosylation and methylation reactions.

From the authors’ perspective, the integration of structural, pharmacological, and biosynthetic insights offers a robust and coherent foundation for future studies focused on metabolic engineering, quality control of herbal preparations, and the exploration of *P. fruticosa* saponins as candidates for pharmaceutical and biotechnological applications.

### 5.2. Phenolic Constituents of Polyscias fruticosa: Chemotaxonomic Significance and Pharmacological Perspectives

#### 5.2.1. Flavonoids and Phenolic Acids in *Polyscias fruticosa*

To date, only one comprehensive review on phenolic compounds in the genus *Polyscias* has been published, authored by Ashmawy et al. [4]. Identified compounds include quercetin-3-O-glucoside (*P. fulva*), luteolin (*P. nodosa*), lichexanthone (*P. fulva*), tamaraxetin 3,7-di-O-α-L-rhamnopyranoside (*P. balfouriana*, *P. guilfoylei*), and various other phenolic derivatives. The 2020 review by Ashmawy et al. [4] did not include phenolic compounds from *P. fruticosa*, as these constituents were only confirmed in subsequent studies published between 2021 and 2023. This section provides, for the first time, a comprehensive summary and discussion of 20 phenolic compounds, encompassing both simple phenolics (e.g., 2,6-dimethoxyphenol) and more complex lignin-derived eugenol-type structures (e.g., sinapyl alcohol, 1′-hydroxyeugenol, 1,2-bis(4-hydroxy-3-methoxyphenyl)ethylene) (Table 2).

Rarison et al. [10] identified nine phenolic compounds in the dichloromethane fraction (PFLD) of *P. fruticosa* leaf extract. After extraction with 95% ethanol and partitioning with n-hexane and dichloromethane, GC-MS analysis revealed several low-polarity phenolics, including 2-methoxy-4-vinylphenol (**36**), 2,6-dimethoxy-4-vinylphenol (**32**), sinapyl alcohol (**39**), 4-allyl-2,6-dimethoxyphenol (**37**), 1′-hydroxyeugenol (31), 2,6-dimethoxyphenol (**33**), and the dimeric 1,2-bis(4-hydroxy-3-methoxyphenyl)ethylene (**38**). Two additional compounds, 4-vinylphenol (**34**) and vanillin (**35**), were also detected; however, their identification remains tentative. These two structures are commonly recognized as potential thermal artifacts or lignin degradation products and are known to occur as contaminants in GC-MS profiles. In the absence of orthogonal validation (e.g., LC–MS or NMR), their biosynthetic origin in *P. fruticosa* remains speculative and should be interpreted with caution. Therefore, their inclusion in the phenolic profile of this species should be treated with caution. These findings suggest that the lipophilic leaf fraction of *P. fruticosa* may represent an underexplored source of structurally unusual phenolic compounds with potential antioxidant and anti-inflammatory activities, warranting further investigation (Table 2).

Selvaraj et al. [21] identified phenolic compounds in the ethyl acetate fraction derived from a 75% ethanol extract of *P. fruticosa* roots (EEPF). Chromatographic purification yielded four phenolic compounds, including one novel and three previously known structures. The newly discovered compound, named politoic acid (**28**), was characterized by HRESI-MS and 1D/2D NMR (^1^H, ^13^C, HSQC, HMBC) as (E)-2-(4-((E)-2-carboxyvinyl)-2-methoxyphenoxy)-3-(4-hydroxyphenyl)acrylic acid—a unique lignin-derived dimer not previously reported in any *Polyscias* species, thereby extending the chemotaxonomic spectrum of this genus.

Additionally, two oligomers were isolated: 8-O-4-dehydrodiferulic acid (**29**) and 8-O-4/8-O-4-dehydrotriferulic acid (**30**), representing a ferulic acid dimer and trimer, respectively, both with known antioxidant and neuroprotective effects. These compounds were identified for the first time in *P. fruticosa* and the *Polyscias* genus overall, substantially expanding its known phenolic repertoire [21].

The same fraction also contained protocatechuic acid (PCA) (**27**), a well-known plant-derived phenolic acid with potent antioxidant activity [86]. Although structurally familiar, this compound had not previously been reported in *P. fruticosa*, making its identification a notable contribution to the species’ phytochemistry and potentially linking it to the documented pharmacological properties of other Araliaceae taxa. PCA acts through ROS scavenging, metal chelation, and inhibition of lipid peroxidation. Furthermore, it exhibits antidiabetic properties by inhibiting α-amylase and α-glucosidase, thereby reducing postprandial hyperglycemia. Notably, it inhibited α-glucosidase with an IC_50_ of 1.76 µM [86,87]. PCA has also shown anticancer activity by suppressing proliferation, inducing apoptosis, and modulating signaling pathways (NF-κB, PI3K/AKT, MAPK), and exerts chemopreventive effects by normalizing redox balance and cellular metabolism [88].

Do et al. [8] conducted a detailed phytochemical study of *P. fruticosa* leaves extracted with 95% ethanol, followed by partitioning with diethyl ether, ethyl acetate, and n-butanol. From the diethyl ether fraction (26 g), obtained from 859 g of dried leaves, nine phenolic compounds were isolated using silica gel and Sephadex LH-20 chromatography.

Among them were two flavonol glycosides, kaempferol-3-O-rhamnoside (**20**) and quercetin-3-O-rhamnoside (**21**), along with one flavanone (liquiritigenin) (**23**), one coumarin (esculetin) (**22**), and one phenolic acid (caffeic acid) (**24**). Additionally, two cinnamic aldehyde derivatives, 4-hydroxycinnamaldehyde (**25**) and (E)-isoferulaldehyde (**26**), were identified. Despite their structural simplicity, these compounds contribute to the overall phenolic content due to their hydroxylated aromatic systems, which suggests that these compounds may play a relevant role in the antioxidant profile of *P. fruticosa*. (Table 2).

Structural elucidation was confirmed by ^1^H and ^13^C NMR and HRESI-MS, supported by a comparison of the available literature. Interestingly, two triterpenoids, 6,15α-epoxy-1β,4β-dihydroxyeudesmane and olean-12-ene-3β,15α-diol, were also isolated for the first time in the *Polyscias* genus. The aldehydic and acidic phenolics identified in this fraction were previously unreported in *P. fruticosa*.

Altogether, these findings significantly expand the known phenolic profile of *P. fruticosa*, particularly regarding its lipophilic constituents. The discovery of novel dimers and oligomers, as well as rare cinnamic derivatives and eugenol-related compounds, underscores the species’ chemical diversity and potential pharmacological relevance. However, further validation using non-volatile analysis methods (e.g., LC-MS, NMR) is recommended, especially for structurally labile or thermally sensitive molecules.

#### 5.2.2. Extraction and Analysis of Phenolic Compound Fractions

In recent years, research on phenolic compounds in *P. fruticosa* has increasingly focused on optimizing extraction protocols and conducting comprehensive phytochemical analyses of plant fractions. Studies have particularly emphasized the role of extraction variables in determining the qualitative and quantitative profile of phenolic metabolites. Particular attention has been paid to evaluating the efficacy of solvents with different polarities, applying assisted extraction techniques, and assessing the influence of physicochemical parameters, including extraction time, temperature, and plant-to-solvent ratio.

Nguyen et al. [89] systematically examined the impact of ethanol concentration (50–90%) on the extraction efficiency of *P. fruticosa* roots.

Using 90% ethanol at 30 °C for 3 h (1:20 ratio), the authors achieved the highest total phenolic and flavonoid contents (96.09 mg GAE·g^−1^ and 58.30 mg QE·g^−1^, respectively), which positively correlated with antioxidant activity (DPPH, ABTS). Building upon these findings, Quoc et al. [59] implemented ultrasound-assisted extraction (UAE), which significantly enhanced TPC and TFC compared to maceration. Under 80% ethanol, 40 °C, and 30 min treatment (300 W), UAE resulted in higher TPC/TFC values and improved antioxidant readouts (DPPH, ABTS, FRAP). These results suggest that UAE provides a more efficient and reproducible alternative for extracting antioxidant phenolics from this species.

Notably, the use of low-polarity solvents such as dichloromethane [10] and diethyl ether [9] enabled the detection of lipophilic compounds, including sinapyl alcohol, eugenol-type dimers, vinylphenols, and ferulic acid oligomers. However, some of these—particularly 4-vinylphenol and vanillin—may represent degradation products or analytical artifacts, rather than genuine biosynthetic metabolites. Therefore, we caution against overinterpretation of these GC-MS-derived compounds without orthogonal structural confirmation (e.g., NMR, LC-MS).

Moreover, in vitro culture experiments conducted by Le et al. [12] demonstrated that elicitation with jasmonic acid, mannitol, and yeast extract significantly enhanced both phenolic biosynthesis and the activity of antioxidant enzymes, such as peroxidase (POD) and catalase (CAT). Although promising, the reproducibility and scalability of such elicitor-driven enhancements require further validation, especially under industrially relevant conditions.

In summary, the phytochemical study of phenolics in *P. fruticosa* benefits from well-designed extraction strategies, especially UAE and solvent polarity gradients. These approaches facilitate the recovery of structurally diverse phenolics. Nonetheless, further chemical verification is required to confirm the natural occurrence of several low-polarity compounds before attributing pharmacological activity or biosynthetic significance.

#### 5.2.3. Pharmacological Significance of Selected Phenolic Compounds Identified in *Polyscias fruticosa*

Phenolic compounds are key contributors to the biological activity of medicinal plants, exhibiting a wide range of pharmacological effects, including antioxidant, anti-inflammatory, antimicrobial, and neuroprotective properties [90]. In *P. fruticosa*, accumulating evidence suggests that these metabolites—comprising both ubiquitous constituents (e.g., quercetin, caffeic acid) and unique lignin-derived derivatives—play a substantial role in the therapeutic effects traditionally associated with this species [4,17,21].

In this section, only selected phenolic compounds with either confirmed structural identity or significant biological activity have been discussed in detail. This selective approach reflects the authors’ intention to emphasize pharmacologically relevant metabolites while avoiding speculative interpretation of tentative or potentially artifactual identifications.

In vitro studies have confirmed strong antioxidant activity in ethanol extracts from *P. fruticosa* roots and leaves, which correlates positively with their phenolic content. Nguyen et al. [89] reported that root extracts exhibited potent radical-scavenging activity against DPPH (IC_50_ = 96.14 µg·mL^−1^) and ABTS (IC_50_ = 38.76 µg·mL^−1^), with total phenolic content (TPC) and total flavonoid content (TFC) being strongly associated with antioxidant potential. Similar results were obtained for leaf extracts by Ly et al. [20], which showed pronounced free radical scavenging activity and inhibition of lipid peroxidation in TBARS assays.

Phenolic compounds isolated from lipophilic fractions, including 1′-hydroxyeugenol (**31**), sinapyl alcohol (**39**), and the dimeric 1,2-bis(4-hydroxy-3-methoxyphenyl)ethylene (**38**), exhibited significant anti-inflammatory activity through inhibition of nitric oxide (NO) production in LPS-stimulated RAW 264.7 macrophages [17]. These findings suggest that lipophilic phenolics may modulate key inflammatory signaling pathways. Its chemical structure, featuring both methoxy and hydroxyl groups on the aromatic ring, confers high antioxidant capacity and facilitates redox regulation [91].

Le Roy et al. [91] showed that sinapyl alcohol is stored in plants as glucosides, which can be mobilized in response to environmental stimuli, providing protection against pathogens and UV radiation.

Further evidence supports the anti-inflammatory and antinociceptive properties of sinapyl alcohol in vivo. Oral administration significantly reduced edema and pain in formalin- and carrageenan-induced inflammation models, highlighting its therapeutic potential in inflammatory and pain-related disorders [92]. Additionally, sinapyl alcohol exhibits moderate antimicrobial activity and regulates lignin biosynthesis by modulating cell wall differentiation, which may underlie its cytotoxic and neuroprotective potential [93].

Ferulic acid derivatives isolated from *P. fruticosa* roots, including 8-O-4-dehydrodiferulic acid (**29**) and 8-O-4/8-O-4-dehydrotriferulic acid (**30**), demonstrated neuroprotective effects in oxidative stress models. Selvaraj et al. [21] attributed these effects to their antioxidant capacity and modulation of apoptotic and intracellular signaling pathways in neuronal cells.

Elicitation with jasmonic acid and other stressors enhanced phenolic biosynthesis and antioxidant potential in in vitro cultures of *P. fruticosa* [12]. This observation suggests that elicitation strategies could be further optimized to boost pharmacologically valuable phenolic content in vitro.

Quercetin derivatives may also contribute to the antimicrobial activity of *P. fruticosa*. A review by Mawea et al. [94] highlighted the antibacterial efficacy of quercetin and quercitrin in other plant models, supporting the ethnomedicinal use of *P. fruticosa* for treating infections and inflammatory skin conditions.

Among the most pharmacologically significant polyphenols in *P. fruticosa* is kaempferol-3-O-α-L-rhamnoside (afzelin) (**20**), isolated from the diethyl ether fraction of leaf extracts [8]. Afzelin exhibits antioxidant, cytoprotective, anti-inflammatory, and anticancer properties [95,96,97,98,99]. In lung cancer models, afzelin induced immunogenic cell death (ICD) by downregulating NAD(P)H: quinone oxidoreductase 2 (NQO2), triggering ER stress, and promoting DAMP release (ATP, HMGB1, calreticulin), thereby stimulating antitumor immune responses [96]. In murine models, afzelin suppressed Ehrlich ascites carcinoma (EAC) proliferation by ~71% at 50 mg·kg^−1^ with minimal toxicity [97]. It also enhanced SPF values in sunscreen formulations and exhibited mild anti-adipogenic activity in 3T3-L1 cells, broadening its potential applications in cosmetics and metabolic health [95,99].

Quercitrin (quercetin-3-O-α-L-rhamnoside) (**21**), another flavonol glycoside identified in the same fraction [8], is known for its antioxidant, anti-inflammatory, and cytoprotective properties. In osteoarthritis models, quercitrin inhibited NF-κB signaling and suppressed matrix metalloproteinases and pro-inflammatory cytokines [100]. In myocardial infarction models, it improved cardiac function by promoting M2 macrophage polarization and enhancing mitochondrial respiration [101]. Quercitrin also improved gut microbiota composition and reduced serum cholesterol in zebrafish models, confirming its multifaceted health benefits [102,103].

Esculetin (6,7-dihydroxycoumarin) (**22**), a coumarin derivative identified for the first time in *P. fruticosa* [8], exhibits anticancer, lipid-lowering, and metabolic regulatory activities [104,105,106,107]. In hepatocellular carcinoma models, it induced ferroptosis by inhibiting the Nrf2–xCT/GPx4 axis and impairing glycolysis through GPI inhibition [104,105,106,107]. Additionally, it promoted HDL formation via CD36 upregulation in adipose macrophages and reduced fat accumulation in *C. elegans* via insulin/IGF-1 and AMPK signaling pathways [105,106].

Caffeic acid (**24**), a hydroxycinnamic acid detected in *P. fruticosa* for the first time [8], exhibits robust antioxidant and anti-inflammatory properties. It scavenges reactive radicals, chelates metal ions, and inhibits pro-inflammatory cytokines via NF-κB and MAPK suppression [108,109,110]. It also exerts anticancer effects, particularly against HCC, by suppressing STAT3 signaling and promoting apoptosis [108].

The identification of such well-known bioactives in *P. fruticosa* contributes to the chemotaxonomic characterization of the species and its alignment with other pharmacologically rich Araliaceae taxa.

4-hydroxycinnamaldehyde (**25**), identified by Do et al. [8], demonstrated antiproliferative and pro-apoptotic effects via Wnt/β-catenin and BAG3/HSF1 pathway inhibition [111,112]. It also suppresses NF-κB activity via ERK modulation, supporting its anti-inflammatory potential [113].

Finally, (E)-isoferulaldehyde (**26**), an aldehydic derivative of ferulic acid, was shown to inhibit NO production in LPS-stimulated macrophages (IC_50_ = 19.0 mg·dm^−3^), and exhibits antimicrobial and cytotoxic effects [114,115]. Its presence in *P. fruticosa* supports its chemotaxonomic classification among phenylpropanoid-rich species and may explain some of its neuroprotective and immunomodulatory effects.

Taken together, this critical analysis of selected phenolic compounds in *P. fruticosa* highlights their diverse pharmacological profiles and supports the ethnomedical relevance of the species. Further integrative studies are needed to confirm their bioactivity in vivo and to validate the most promising compounds as lead candidates for therapeutic development.

### 5.3. Sterols and Steroidal Derivatives Identified in Polyscias fruticosa

Sterols are fundamental components of plant cell membranes, contributing to membrane fluidity, permeability, and receptor-mediated signaling. In medicinal plants, phytosterols are valued for their roles in modulating inflammation, oxidative stress, and lipid metabolism [116,117].

In *P. fruticosa*, recent phytochemical investigations have confirmed the presence of sterols and steroidal derivatives, enhancing the known chemical diversity of the species (Table 3). Rarison et al. [10] identified two sterols, stigmasterol (**40**) and (3β,5α)-stigmasta-7,16-dien-3-ol (**41**), in both the n-hexane (PFLH) and dichloromethane (PFLD) leaf extracts. The identification of these sterols in both moderately and strongly non-polar fractions confirms their high lipophilicity and supports their accumulation in membrane-rich tissues.

Additionally, Nguyen et al. [118] isolated an unusual sterol, 22-dehydro-24-isopropylcholesterol (**42**), from petroleum ether–diethyl ether (1:1, *v*/*v*) root extracts (Table 3). This sterol, previously described mainly in marine taxa, constitutes a rare occurrence in terrestrial flora, suggesting that *P. fruticosa* may possess biosynthetic traits not yet fully characterized within Araliaceae.

Earlier reviews by Ashmawy et al. [4] documented the presence of β-sitosterol and 3-O-β-D-glucopyranosyl-sitosterol in other *Polyscias* species, such as *P. fulva*, *P. nodosa*, and *P. guilfoyeli* [4,119,120]. However, their presence in *P. fruticosa* was not confirmed until the reports by Rarison et al. [10] and Nguyen et al. [118]. The confirmation of these sterols in *P. fruticosa* aligns with previous reports in congeneric species and reinforces its position within a phytochemically rich lineage. From a chemotaxonomic perspective, the diversity of sterols in *P. fruticosa* supports its classification among phytosterol-producing Araliaceae and provides a foundation for comparative biosynthetic studies.

From the authors’ perspective, the current state of research on sterols in *Polyscias fruticosa* remains limited, particularly in comparison to the extensive studies on triterpenoid saponins and polyphenols. Although several phytosterols have been identified, including rare or unusual compounds such as 22-dehydro-24-isopropylcholesterol (**42**), their biosynthetic origins, tissue-specific distribution, and regulatory mechanisms have not been elucidated. Moreover, the lack of in vivo studies hinders a comprehensive understanding of their pharmacological roles, including potential synergistic effects with other lipophilic metabolites. Further investigations should aim to characterize the sterol biosynthetic pathway, assess the bioavailability of individual compounds, and explore their therapeutic relevance through integrative pharmacological models. Such studies would clarify the contribution of sterols to the traditional and modern medicinal use of *P. fruticosa* and enhance the rationale for their inclusion in phytopharmaceutical formulations.

#### Biological Properties of Sterols and Steroidal Derivatives in *Polyscias fruticosa*

*Polyscias fruticosa* contains a diverse array of sterolic constituents with distinct structural features and pharmacological implications. Among them, stigmasterol, detected in both n-hexane and dichloromethane extracts, is the most thoroughly studied [4]. Stigmasterol (**40**) is known for its anti-inflammatory, antioxidant, antidiabetic, and anticancer effects [118,119,120]. Its anti-inflammatory activity involves suppression of cyclooxygenase-2 (COX-2) and inducible nitric oxide synthase (iNOS), inhibition of NF-κB translocation, and downregulation of pro-inflammatory cytokines including TNF-α, IL-1β, and IL-6.

The antioxidant effects result from both direct ROS scavenging and induction of endogenous antioxidant enzymes such as catalase, superoxide dismutase (SOD), and glutathione peroxidase (GPx).

Anticancer effects are mediated through modulation oncogenic signaling via inhibition of Akt/mTOR and JAK/STAT pathways and downregulation of anti-apoptotic proteins such as Bcl-2 and Bcl-xL [119,120].

(3β,5α)-Stigmasta-7,16-dien-3-ol (**41**), a less commonly reported Δ^7^-sterol with double bonds at C-7 and C-16, was also identified in *P. fruticosa* [10]. Although direct pharmacological data on this compound are limited, structurally similar Δ^7^-sterols have demonstrated membrane-stabilizing, antifungal, and cytostatic properties [116]. Their rigid steroidal structure is thought to support membrane integrity under oxidative and osmotic stress. The co-occurrence of this sterol with stigmasterol in lipophilic extracts may imply cooperative effect in modulating membrane fluidity and lipid raft-associated signaling.

Of particular interest is 22-dehydro-24-isopropylcholesterol (**42**), an atypical marine-type sterol isolated from the roots of *P. fruticosa* [118]. This sterol, characterized by a double bond at C-22 and an isopropyl group at C-24, is rarely found in terrestrial plants. In marine systems, related sterols have demonstrated cytotoxic, antimicrobial, and immunomodulatory effects, while its biological activity in *P. fruticosa* has not been investigated, its structural similarity to marine sterols suggests potential bioactivity, including membrane disruption, apoptosis induction in cancer cells, and inhibition of microbial enzymes. Its presence in *P. fruticosa* suggests a unique biosynthetic route and warrants further study as a potentially bioactive and taxonomically significant metabolite.

Altogether, the sterolic profile of *P. fruticosa*—comprising stigmasterol, (3β,5α)-stigmasta-7,16-dien-3-ol, and 22-dehydro-24-isopropylcholesterol—underscores its biochemical complexity and value as a source of bioactive natural products. These compounds likely contribute synergistically to the modulation of inflammation, oxidative stress, and cellular homeostasis.

From the authors’ perspective, their diversity highlights the need for in-depth studies on biosynthesis, subcellular distribution, and functional activity. Their structural features also make them promising lead scaffolds for further drug development [10].

### 5.4. Essential Oils and Volatile Constituents Identified in Polyscias fruticosa

Studies on the essential oils of *P. fruticosa* have revealed a complex composition of terpenoid constituents, with marked variation depending on the extraction method employed. Comparative analysis of n-hexane and dichloromethane extracts has demonstrated distinct chemical profiles, with specific classes of terpenoids predominating in each fraction (Table 4) [10].

The n-hexane extract, obtained by maceration, was notably enriched in hydrocarbon sesquiterpenes, including β-caryophyllene (**57**), germacrene D (**55**), α-humulene (**64**), and α-ylangene (**66**). These lipophilic compounds occurred in high concentrations and are known for their anti-inflammatory properties and potential modulatory effects on the endocannabinoid system.

In contrast, the dichloromethane extract was characterized by a higher content of oxygenated sesquiterpenes, notably spathulenol, caryophyllene oxide (**73**), and globulol, which have been associated with antioxidant, cytotoxic, and topical anti-inflammatory activities. This fraction also contained structurally distinct terpenoids such as ledol (**83**) and (4S,5R)-5-hydroxycarvophyll-8(13)-ene-4,12-epoxide (**78**), compounds of interest for their potential roles in redox modulation [10].

Comparative profiling of both fractions revealed that the n-hexane fraction is dominated by hydrocarbon sesquiterpenes with pronounced lipophilicity and potential activity at CB2 receptors and pro-inflammatory signaling pathways, whereas the dichloromethane extract was richer in oxygenated sesquiterpenes with stronger antioxidant and antiproliferative potential [10]. These observations suggest that the pharmacological effects of *P. fruticosa* extracts may be selectively enhanced through targeted extraction strategies, emphasizing the need for receptor-level studies to validate their mechanisms of action.

These compositional differences reflect solvent polarity and selectivity: n-hexane preferentially extracts nonpolar hydrocarbon terpenoids, while dichloromethane, with moderate polarity, facilitates the extraction of more functionally diverse and oxygenated constituents. Such differentiation is crucial, as the lipophilic and oxygenated fractions are likely to elicit distinct biological responses depending on their structural features. These findings underscore the critical importance of extraction methodology in shaping the chemical and pharmacological profiles of *P. fruticosa* preparations.

From the authors’ perspective, the presence of sesquiterpenes with documented affinity for CB2 and TRPV1 receptors, such as β-caryophyllene and caryophyllene oxide, lends strong support to the ethnomedicinal use of *P. fruticosa* in inflammatory and pain-related conditions. Although preliminary in vitro and in silico studies are promising [10], we emphasize the need for in vivo validation, pharmacokinetic characterization, and mechanistic analyses to substantiate these bioactivities. In particular, sesquiterpenes with lipophilic properties and receptor-binding potential represent attractive candidates for development into topical or transdermal formulations, aligning with the traditional modes of administration.

The foundational study by Brophy et al. [9] provided one of the first comprehensive GC-MS analyses of essential oils distilled from *P. fruticosa* leaves collected in Fiji and Thailand. Hydrodistillation of the leaf material yielded approximately 24 sesquiterpenes, among which β-elemene (**60**), α-bergamotene (**52**), germacrene D (**55**), and (E)-γ-bisabolene (**51**) were predominant. Other constituents included α-humulene (**64**), β-caryophyllene (**57**), and aromadendrene (**63**), as well as trace amounts of monoterpenes (e.g., limonene) (**43**) and low-molecular-weight alcohols such as 1-hexanol (**45**) and (Z)-hex-3-en-1-ol (**46**) (Table 4) [9].

Importantly, Brophy et al. [9] observed considerable qualitative and quantitative variation in oil composition depending on geographical origin. For example, oxygenated sesquiterpenes accounted for ~20% in the Bangkok I sample, were nearly absent in the Bangkok II sample, and reached intermediate levels (~7%) in the Fiji sample. These differences may be attributed to environmental and genetic factors, as well as to chemical transformations such as isomerization or oxidation induced by light or heat during distillation. The detection of sesquiterpene isomers, including β-bourbonene (**68**) and δ-cadinene (**65**), may reflect such degradation processes. In light of these concerns, we support the future application of orthogonal techniques such as NMR or GC×GC to confirm compound identity and eliminate possible artifacts.

Whereas Brophy et al. [9] focused on hydrodistilled essential oils, Rarison et al. [10] extended the analysis to include solvent-extracted fractions, thereby broadening the understanding of the chemical variability and potential pharmacological significance of *P. fruticosa* terpenoids. The combination of hydrodistillation and solvent extraction techniques allows for a more complete characterization of both volatile and semi-volatile constituents with differing biological potential.

From the authors’ perspective, the sesquiterpene-rich essential oil profile of *P. fruticosa* demonstrates high pharmacological potential, especially for compounds such as β-caryophyllene, caryophyllene oxide, and germacrene D. Due to their lipophilicity and documented bioactivities, these metabolites warrant further investigation as candidates for topical or transdermal therapies. Standardization efforts should also account for chemical variability linked to environmental and genetic factors. To ensure compound authenticity and uncover novel activities, advanced analytical methods such as NMR or GC×GC are recommended.

### 5.5. Pharmacologically Relevant Rare Terpenoids and Norisoprenoids Identified in Polyscias fruticosa

*Polyscias fruticosa* is recognized for its complex phytochemical profile, which includes numerous structurally unique compounds with potential pharmacological applications. In addition to the commonly reported sesquiterpenes and monoterpenes, advanced gas chromatography–mass spectrometry (GC-MS) analyses have revealed the presence of several rare and structurally distinctive terpenoids and norisoprenoids in *P. fruticosa*.

Although many of these constituents occur in low abundance, their structural uniqueness and bioactivities—also documented in other species—suggest potential relevance in pharmacological or ecological contexts.

However, the possibility that some volatile constituents may result from analytical artifacts or thermal degradation during GC-MS analysis should be taken into account. Therefore, conclusions based solely on GC-MS data require confirmation by complementary analytical techniques such as NMR or LC-MS to ensure structural accuracy. Despite their minor abundance, these metabolites may contribute substantially to the plant’s biological activity and adaptive responses to environmental stressors.

Among these newly identified compounds is loliolide (**49**), a norisoprenoid lactone known for its potent antioxidant, anti-inflammatory, and anti-aging properties. While loliolide is commonly found in marine algae [121] and was recently confirmed in *Camellia ptilosperma* leaves [122], its detection in *P. fruticosa* is uncommon. This suggests possible chemotypic variability or previously unrecognized biosynthetic pathways within the Araliaceae family. Loliolide efficiently scavenges reactive oxygen species (DPPH, H_2_O_2_), protects cells against oxidative damage, and exerts cytoprotective effects in both in vitro and in vivo models (including Vero cells and zebrafish embryos) [123]. It also acts as a natural herbivore resistance inducer, functioning independently of the jasmonic acid pathway [124], and enhances dermal papilla cell activity via AKT/β-catenin signaling [125]. Recent studies further highlight its neuroprotective properties against glutamate-induced excitotoxicity, mediated through the MAPK/Nrf2 pathway and improved mitochondrial function [126]. These diverse effects render loliolide a promising multipotent molecule that may contribute to both the ecological and pharmacological profile of *P. fruticosa*.

Actinidiolide (**48**), another norisoprenoid lactone detected in *P. fruticosa*, is a degradation product of carotenoids and shares structural features with loliolide. While its specific activity has not yet been confirmed in *P. fruticosa*, studies in other species suggest its involvement in cytoprotective and antioxidant pathways. Its presence further supports the relevance of norisoprenoids in the chemotaxonomic and ecological profile of the species [10].

Alismol (**75**), an oxygenated sesquiterpene originally isolated from *Alisma orientale* and later reported in *Vladimiria souliei* [127], has also been identified in *P. fruticosa*. This compound displays notable anti-inflammatory activity via activation of the Nrf2 pathway and inhibition of pro-inflammatory mediators, including TNF-α, IL-1β, IL-6, and COX-2 expression [128,129]. In LPS-stimulated microglial cells, alismol suppresses nitric oxide (NO) and prostaglandin E_2_ (PGE_2_) production by inhibiting NF-κB signaling while sparing MAPK pathways [127]. Additionally, it exhibits neuroprotective effects by mitigating microglia-mediated neuronal cytotoxicity and alleviating lung injury in LPS-induced inflammatory models [128,129].

α-bergamotene (**52**), a hydrocarbon sesquiterpene detected in the essential oil of *P. fruticosa*, has been reported in other plant species such as *Guarea cedrata* and *Schisandra perulata* [130,131]. It demonstrates a moderate antioxidant capacity and exhibits antibacterial and antifungal activities against *Escherichia coli*, *Salmonella typhimurium*, and *Candida albicans*, thereby contributing to the antimicrobial potential of *P. fruticosa* extracts [132,133].

Another notable component is α-gurjunene (**70**), a hydrocarbon sesquiterpene identified in the n-hexane fraction of *P. fruticosa* leaf extracts. The presence of α-gurjunene significantly enhances both the pharmacological and aromatic profiles of the essential oil. Studies in other species, such as *Araucaria columnaris* [134] and *Globba schomburgkii* [135], have shown that α-gurjunene exhibits strong antibacterial and antifungal properties, supporting its relevance in plant defense mechanisms.

It is noteworthy that α-gurjunene, together with other sesquiterpenes such as β-caryophyllene (**57**) and caryophyllene oxide (**73**), may act synergistically to enhance the antioxidant and antimicrobial properties of *P. fruticosa* extracts. Their abundance in lipophilic fractions (n-hexane and dichloromethane) suggests their potential applicability in phytotherapeutic formulations and nutraceutical development [134,135].

Of particular interest is 6,10,14-trimethylpentadecan-2-one (**79**), a C18 isoprenoid ketone derived from the oxidative degradation of phytol (**87**). This compound may result from abiotic breakdown of (E)-phytenal or from the hydrolysis of photochemically generated phytyl-chain derivatives of chlorophyll [136]. Its detection in *P. fruticosa* indicates possible environmental influences on the plant’s metabolome and highlights how endogenous metabolism can interact with ecological conditions. However, as this compound can be a photodegradation product, its presence should be interpreted with caution in the context of GC-MS-based metabolite profiling.

In summary, these rare terpenoids and norisoprenoids highlight the importance of comprehensive chemical profiling in *P. fruticose*. Among them, loliolide (**76**) stands out for its multifunctional activities, including antioxidant and anti-inflammatory effects [123,137], induction of herbivore resistance [124], stimulation of dermal papilla cell activity [125], and neuroprotective effects [126]. Similarly, alismol (**71**), α-bergamotene (**48**), α-gurjunene (**66**), and 6,10,14-trimethylpentadecan-2-one (**79**) contribute to the plant’s potential therapeutic spectrum, particularly its anti-inflammatory, antioxidant, antimicrobial, and ecological defense properties. Future studies should aim to confirm the occurrence of these compounds using orthogonal analytical tools and to evaluate their synergistic interactions with major bioactive constituents of *P. fruticosa*.

### 5.6. First Identification and Biological Role of Tocopherols in Polyscias fruticosa

According to the comprehensive phytochemical review by Ashmawy et al. [4], there had been no previous evidence confirming the presence of tocopherols (vitamin E and its derivatives) (Table 5) in any species of the genus *Polyscias*. The authors cataloged a wide range of phytochemical constituents, such as triterpenoid saponins, terpenoids, and sterols, but tocopherols were notably absent. This observation supports the novelty and taxonomic importance of recent findings by Rarison et al. [10], who reported, for the first time, the occurrence of tocopherols in *P. fruticosa*, specifically within the n-hexane fraction of leaf extracts. This finding represents a notable expansion of the known metabolomic diversity of the species and underscores the importance of including lipophilic fractions in phytochemical screening.

Tocopherols, particularly α-tocopherol, are well-established lipid-soluble antioxidants in both plant and animal systems [138]. Their principal function has traditionally been attributed to the scavenging of reactive oxygen species (ROS) and the protection of membrane lipids from peroxidation. However, emerging evidence indicates that α-tocopherol also exerts biological functions independent of its antioxidant properties. These include modulation of gene expression, regulation of intracellular signaling cascades, and involvement in lipid metabolism [138,139]. From a phytopharmacological perspective, such pleiotropic effects may enhance the adaptogenic potential of *P. fruticosa* and partially explain its traditional use in managing oxidative stress and inflammation.

The detection of tocopherols in *P. fruticosa* may also reflect their physiological role in stabilizing membrane structures and influencing lipid raft composition (Table 5). Tocopherols are known to interact with phospholipids and sterols in the lipid bilayer, potentially affecting membrane fluidity and signal transduction under stress conditions [138]. This membrane-level modulation may act synergistically with sterols previously identified in *P. fruticosa*, suggesting coordinated functions of lipophilic compounds in the plant’s response to abiotic stress. Furthermore, metabolites of tocopherols, including carboxychromanols and CEHCs (2-(β-carboxyethyl)-6-hydroxychromans), have demonstrated a range of bioactivities, such as anti-inflammatory, antiproliferative, and cytoprotective effects in mammalian systems [139]. Although these metabolites have not yet been reported in *P. fruticosa*, their biosynthetic plausibility invites targeted metabolomic studies.

In our opinion, the functional role of tocopherols in *P. fruticosa* warrants further investigation not only due to their pharmacological relevance, but also their potential contribution to stress resilience and chemotaxonomic distinction within Araliaceae.

This first report of tocopherols in the *Polyscias* genus emphasizes the importance of detailed phytochemical investigation and invites further study into their functional roles in plant physiology and therapeutic potential. Future research should focus on quantification, localization, and possible synergistic interactions of tocopherols with other lipophilic constituents in *P. fruticosa*, such as sesquiterpenes, diterpenes, and triterpenoids. Such investigations may shed light on the integrative defense mechanisms of the plant and support its potential for phytopharmacological and nutraceutical applications.

### 5.7. Bioactive Polyacetylenes in Polyscias fruticosa: Phytochemical Identification and Pharmacological Relevance

Polyacetylenes are a class of unsaturated aliphatic compounds characterized by the presence of one or more carbon–carbon triple bonds. In *P. fruticosa*, these metabolites constitute a pharmacologically significant group of secondary metabolites, exhibiting a broad range of biological activities, including anti-inflammatory, cytotoxic, antimicrobial, and immunomodulatory effects (Table 6) [11,132,133,140].

The presence of polyacetylenes in *P. fruticosa* was initially suggested by earlier studies referenced by Do et al. [7], which reported their occurrence in the roots and associated them with the plant’s traditional use in treating inflammatory conditions. Subsequent phytochemical investigations confirmed the presence of four principal polyacetylenes, namely falcarinol (**92**) (also known as panaxynol), falcarindiol, heptadeca-1,8-diene-4,6-diyne-3,10-diol (**93**), and heptadeca-1,8-diene-4,6-diyne-3-ol-10-one (**94**), in both root and leaf tissues. Rarison et al. [10] further expanded the understanding of polyacetylene distribution in *P. fruticosa* by identifying falcarinol (**92**) and hexadeca-5,7,9,11-tetrayne-1,16-diol (**95**) in different solvent fractions of the leaf extract. Falcarinol was predominantly present in the n-hexane fraction (PFLH), consistent with its high lipophilicity, while hexadeca-5,7,9,11-tetrayne-1,16-diol was selectively detected in the dichloromethane fraction (PFLD) at a concentration of 0.94 ± 0.01%. These findings indicate solvent-dependent extraction efficiency and suggest differential tissue distribution and solubility of polyacetylenes within the species. Such solvent-specific activity supports the importance of fractionation strategies in metabolite profiling and bioactivity correlation.

Falcarinol (**92**), a monoacetylene alcohol, has been extensively studied for its pharmacological potential. It exhibits strong anti-inflammatory activity by downregulating inducible nitric oxide synthase (iNOS) and cyclooxygenase-2 (COX-2), suppressing nuclear factor-κB (NF-κB) activation, and reducing the secretion of pro-inflammatory cytokines such as TNF-α and IL-6 [141]. In vitro cytotoxicity has also been reported across diverse cancer cell lines, primarily through oxidative stress and apoptosis-related pathways. However, despite promising preclinical outcomes, its translation to clinical models remains unexplored.

The antimicrobial activity of falcarinol has also been well documented. Lutomski et al. [11] reported its activity against Gram-positive bacteria (*Staphylococcus aureus*, *Streptococcus pyogenes*) and fungal strains (*Candida albicans*, *Microsporum gypseum*), suggesting its relevance in the treatment of infections. Additionally, Rarison et al. [10] demonstrated that falcarinol modulates immune responses by downregulating costimulatory molecules on antigen-presenting cells and suppressing T-cell proliferation in inflammatory environments, supporting its immunomodulatory role.

Mechanistic studies by Alfurayhi et al. [140] have further elucidated the multi-targeted pharmacological profile of falcarinol-type polyacetylenes. These compounds were shown to inhibit iNOS and COX-2 expression, suppress pro-inflammatory cytokine production, activate the Nrf2-mediated antioxidant defense system, and modulate key signaling pathways such as PI3K/Akt and MAPK. This pleiotropic mode of action may underlie their efficacy in both inflammatory and tumorigenic settings.

Neuroprotective effects—though preliminary—have been linked to calcium channel inhibition and mitochondrial preservation under oxidative stress [132]. However, further studies are needed to establish their pharmacodynamic profile and therapeutic index.

Polyacetylenes, especially falcarinol, emerge as promising multifunctional agents in *P. fruticosa*, with reported anti-inflammatory, cytotoxic, antimicrobial, immunomodulatory, and neuroprotective activities. However, compared to triterpenoid saponins or polyphenols, this class remains underexplored. Future studies should prioritize their biosynthetic origins, stability under extraction conditions, and potential synergistic interactions with other lipophilic compounds. Bridging phytochemical data with pharmacodynamic endpoints will be essential to assess their translational potential in phytomedicine.

### 5.8. Fatty Acids in Polyscias fruticosa and Their Pharmacological Significance

Phytochemical investigations of *P. fruticosa* have revealed a diverse composition of fatty acids (FAs), identified by gas chromatography–mass spectrometry (GC-MS) in both the n-hexane (PFLH) and dichloromethane (PFLD) leaf extract fractions [10]. A total of 14 distinct fatty acid-related compounds were identified across both fractions, with 13 compounds detected in the PFLH and 8 in the PFLD. This fraction-dependent distribution reflects the solvent-selective extraction efficiency, where the nonpolar solvent (*n*-hexane) favors the recovery of saturated and unsaturated lipids with low polarity, while dichloromethane, exhibiting intermediate polarity, enables the extraction of moderately polar constituents (Table 7) [10]. Compounds were identified by comparing the resulting spectra with NIST and Wiley libraries [10]. In our view, the differential presence of fatty acids in PFLH and PFLD extracts underscores the influence of solvent polarity on the lipidomic profile of *P. fruticosa*, which may have implications for bioavailability and formulation strategies in phytopharmaceutical development.

In the hexane fraction, saturated FAs such as palmitic acid (**102**, 10.72%) and stearic acid (**103**, 2.28%) were among the most abundant, alongside unsaturated FAs including linolenic acid (**100**, 7.73%), linoleic acid (**99**), and 10-trans,12-cis-linoleic acid (**97**, 8.15%). A particularly notable compound, 9,11-octadecadienoic acid (**96**), a rare positional isomer of linoleic acid, was exclusively identified in the PFLH at 0.24% [10]. Its presence in *P. fruticosa*, not previously reported in the Araliaceae family, may hold chemotaxonomic relevance and suggests that this species biosynthesizes unique lipid mediators with potential therapeutic value [142]. This observation underscores the importance of minor lipid constituents in the pharmacological profiling of medicinal plants.

By contrast, the PFLD displayed a simplified FA profile with lower relative abundances of linolenic acid (2.56%), 10-trans,12-cis-linoleic acid (2.85%), and palmitic acid (5.04%) [10]. However, it contained monoacylglycerol derivatives, including 2-linoleoylglycerol (**98**) and 2-palmitoylglycerol (**108**), possibly resulting from partial hydrolysis or esterification processes. These compounds may participate in lipid signaling pathways and warrant further investigation regarding their metabolic roles. The observed differences between PFLH and PFLD reinforce the critical influence of extraction polarity on the fatty acid composition and reflect the physicochemical diversity of these metabolites in planta.

Among the unsaturated fatty acids, conjugated linoleic acid (CLA)—especially the 10-trans,12-cis isomer—stands out due to its multifaceted pharmacological effects. Recent studies have demonstrated its ability to modulate lipid and glucose homeostasis through activation of PPARγ and PPARδ pathways, upregulation of β-oxidation enzymes such as CPT1A, and increased expression of stearoyl-CoA desaturase (SCD) [143]. Additionally, CLA reduces intracellular triglyceride accumulation and influences fatty acid-binding proteins (FABP1, FABP6), while enhancing short-chain fatty acid (SCFA) production via modulation of the gut microbiome, contributing to improved systemic metabolic function. Special attention should be directed to 9,11-octadecadienoic acid (**96**) due to its dual antioxidant and anti-inflammatory activity. Mechanistic studies have shown that this isomer suppresses COX-2 expression, reduces pro-inflammatory cytokines such as IL-6 and TNF-α, and activates PPARγ-dependent lipid-regulating pathways [144]. It also upregulates the Nrf2/HO-1 antioxidant defense axis and enhances glutathione reductase activity, offering cellular protection against oxidative stress [145]. Related conjugated dienes, such as 13-KODE ((9Z,11E)-13-oxooctadeca-9,11-dienoic acid), exert similar anti-inflammatory effects via inhibition of NF-κB/MAPK signaling and activation of Nrf2 [146].

In our view, the identification of rare fatty acids such as 9,11-octadecadienoic acid positions *P. fruticosa* as a potential source of specialized lipid mediators beyond nutritional relevance. These metabolites may contribute to the anti-inflammatory and metabolic regulatory effects traditionally attributed to this species. However, the lack of in vivo validation, bioavailability data, and mechanistic studies remains a major gap in current research. Future investigations should prioritize the pharmacokinetics, metabolic stability, and synergistic interactions of these fatty acids—particularly in comparison with other Araliaceae taxa—to substantiate their potential for phytotherapeutic or nutraceutical applications.

### 5.9. Newly Identified Volatile and Lipophilic Constituents in Polyscias fruticosa

Recent phytochemical investigations of *P. fruticosa* leaf extracts have led to the identification of several volatile and lipophilic metabolites previously unreported within the *Polyscias* genus [4]. These compounds were characterized by gas chromatography–mass spectrometry (GC-MS) in two solvent-derived fractions: n-hexane (PFLH) and dichloromethane (PFLD) [10]. In total, five newly identified constituents were detected, with the majority either exclusively present or more abundant in the PFLD fraction, highlighting the influence of solvent polarity on metabolite recovery (Table 8).

The presence of unique volatile and lipophilic compounds in PFLD highlights the underexplored chemical space of *P. fruticosa*, especially with regard to less polar but pharmacologically active constituents that may escape detection in aqueous or ethanol-based extracts.

Among these, 3-hydroxy-4,5-dimethylfuran-2(5H)-one (**110**), a furanone-type lactone, stands out due to its known antioxidant and antimicrobial activities. Detected in the PFLD at 0.95 ± 0.03%, this semi-volatile compound may contribute significantly to the pharmacological properties of *P. fruticosa* extracts. Although this compound has been reported in other medicinal plants, its occurrence in *P. fruticosa* may reflect species-specific stress-related biosynthetic pathways that deserve further study.

Additionally, four aliphatic hydrocarbon-related compounds were identified: octanal (**111**), hexadecanal (**112**), 3-methylene-7,11-dimethyl-1-dodecene (**113**), and linoleyl alcohol (**114**) (Table 8). Octanal, a short-chain aldehyde with a citrus aroma, exhibits potent antibacterial and cytotoxic effects, with IC_50_ values below 20 µg·mL^−1^ against selected cancer cell lines [147,148]. Hexadecanal (palmitaldehyde), a saturated long-chain aldehyde, is a known lipid peroxidation byproduct and sphingolipid metabolism intermediate, linked to oxidative stress mechanisms and inflammatory responses relevant to vascular health [148,149].

3-methylene-7,11-dimethyl-1-dodecene (**113**), an unsaturated hydrocarbon likely of terpenoid origin, may act as a semiochemical involved in plant–environment interactions, particularly in defense signaling. In turn, linoleyl alcohol (**114**), a long-chain unsaturated fatty alcohol, plays a role in the structural organization of plant cuticular waxes and membranes. In other botanical systems, it has demonstrated cytoprotective and membrane-stabilizing properties, supporting its potential bioactivity in *P. fruticosa* [149].

The identification of 3-methylene-7,11-dimethyl-1-dodecene and linoleyl alcohol, although not confirmed by isolation or reference standards, suggests that *P. fruticosa* synthesizes functionally diverse lipophilic compounds that may participate in both structural and signaling functions.

The predominance of these compounds in the PFLD fraction emphasizes the efficiency of medium-polarity solvents in isolating structurally diverse lipophilic metabolites. Their absence in previous reports such as that by Ashmawy et al. [4] may reflect either differences in extraction and detection methods or genuine chemotaxonomic distinctiveness. Caution is warranted before assuming novelty, and targeted confirmation using authentic standards and tandem MS is recommended.

Moreover, given the documented biological activities of structurally analogous compounds—particularly octanal and hexadecanal—these newly identified metabolites warrant further pharmacological investigation to evaluate their contributions to the therapeutic potential of *P. fruticosa* extracts.

The lack of functional assays for these lipophilic constituents remains a critical limitation. Future studies should integrate isolation-guided bioassays and metabolomic comparisons across Araliaceae species to validate their relevance in inflammation, oxidative stress, and membrane-associated signaling.

### 5.10. Newly Identified Structurally Diverse Compounds in Polyscias fruticosa

Comprehensive GC-MS analysis of *P. fruticosa* leaf extracts, as performed by Rarison et al. [10], led to the identification of several structurally diverse metabolites that appear to be previously unreported in this species or in the *Polyscias* genus as a whole, based on comparison with the phytochemical inventory by Ashmawy et al. [4]. These constituents, tentatively grouped as “other compounds” due to their chemical heterogeneity, were detected in either the dichloromethane (PFLD) or hexane (PFLH) fractions (Table 9), indicating that untargeted metabolomic profiling can uncover minor, but structurally significant, constituents. Notably, 4,3′-difluoro-4′-methoxybiphenyl was detected exclusively in the PFLH fraction, indicating that nonpolar solvents facilitate the isolation of halogenated aromatic compounds from lipophilic matrices.

Among the identified metabolites, malic acid (**117**) and 4-ethoxy-4-oxobutanoic (**115**) acid are low-molecular-weight carboxylic acids of potential metabolic and antioxidant relevance. Malic acid, a central intermediate of the tricarboxylic acid (TCA) cycle, may serve as a marker of active primary metabolism and intracellular pH regulation. In contrast, the presence of levoglucosan (**118**), a 1,6-anhydrosugar associated with the pyrolytic degradation of lignocellulosic biomass, could reflect oxidative stress responses or senescence-related remodeling of plant cell walls. However, it should be noted that levoglucosan is also a known thermal degradation product, and its detection in GC-MS analysis may represent an analytical artifact rather than a true endogenous metabolite. This highlights a key limitation of GC-MS-based profiling and warrants further validation using non-thermal extraction and detection methods. A particularly noteworthy finding is the detection of 2,3-dihydrothiophene (**116**), a sulfur-containing heterocycle rarely observed in higher plants. Its ecological roles may include allelopathic interactions or microbial defense. Also of significant interest is indole-3-carboxaldehyde (**120**), a tryptophan-derived indolic aldehyde with demonstrated antibacterial and anti-inflammatory activities. This compound is a known natural agonist of the aryl hydrocarbon receptor (AhR), a transcriptional regulator involved in mucosal immunity and epithelial barrier function [141,150]. Recent studies have shown that enteric formulations of indole-3-carboxaldehyde alleviate colitis and modulate host–microbiota interactions through AhR-dependent mechanisms [151]. Furthermore, hydrazone derivatives of this molecule exhibit marked antimicrobial activity against *Staphylococcus aureus* and *Candida albicans*, underscoring its potential as a bioactive lead structure [141]. Its occurrence in *P. fruticosa* suggests a broader distribution of AhR-active indoles within Araliaceae and highlights the need to investigate immunomodulatory effects of this species beyond the currently described anti-inflammatory activity.

The identification of 4,3′-difluoro-4′-methoxybiphenyl (**119**), a halogenated biphenyl derivative, is also of interest. This compound has demonstrated moderate antibacterial activity in vitro [152]. While fluorinated aromatics are often associated with anthropogenic origins or environmental contamination, their presence in *P. fruticosa* may reflect either an unrecognized endogenous biosynthetic route or biotransformation capability inherent to the plant. Alternatively, these compounds might arise from environmental uptake or microbial symbiosis, emphasizing the importance of discriminating endogenous metabolites from external contaminants.

Lastly, 4,6,6-Trimethyl-2-(3-methylbuta-1,3-dienyl)-3-oxatricyclo[5.1.0.0(2,4)]octane (**121**), a polycyclic oxygenated hydrocarbon, further exemplifies the chemical complexity of *P. fruticosa*. Its structural features suggest a potential terpenoid origin and may contribute to the plant’s pharmacological or ecological interactions. However, its precise biosynthetic origin remains unclear and deserves further structural elucidation and biosynthetic pathway analysis.

From a critical perspective, the identification of these structurally diverse and often uncommon compounds raises several key points. First, their detection underscores the importance of fractionated extraction and non-targeted GC-MS profiling in revealing low-abundance phytochemicals. Second, the bioactivity of several identified molecules—or their close analogs—supports the hypothesis that *P. fruticosa* harbors unexplored pharmacologically relevant chemistry. Third, some constituents, such as levoglucosan or fluorinated aromatics, may be artifacts or of exogenous origin, highlighting the need for rigorous methodological controls and confirmatory studies.

Collectively, these newly identified compounds broaden the phytochemical landscape of *P. fruticosa*, reinforce its chemotaxonomic uniqueness within Araliaceae, and suggest its potential as a source of bioactive and structurally novel natural products. Future investigations should focus on confirming the endogenous origin of these metabolites, elucidating their biosynthetic pathways, and evaluating their pharmacological relevance through isolation and bioassay-guided studies.

### 5.11. Critical Review and Future Directions in the Phytochemical Research of Polyscias fruticosa

The phytochemical investigation of *Polyscias fruticosa* has significantly advanced in recent years, shifting from descriptive cataloging to more mechanistically driven exploration of bioactive constituents. The current review integrates both historical and newly identified compounds, highlighting the species’ remarkable chemical diversity and its relevance to ethnopharmacology and drug discovery.

A critical finding is the first confirmed occurrence of tocopherols within the *Polyscias* genus, as reported by Rarison et al. [10]. This compound group was absent from earlier comprehensive reviews, including Ashmawy et al. [4]. This observation demonstrates the added value of revisiting known species with optimized solvent systems and modern GC-MS workflows. Tocopherols, known for their antioxidant and anti-inflammatory roles, suggest that *P. fruticosa* may contribute to redox balance not only through polyphenols and saponins, but also via lipophilic antioxidants. Nonetheless, their relative abundance in extracts is low, and their physiological relevance remains speculative without targeted quantification and in vivo confirmation.

Similarly, the identification of rare fatty acid isomers, notably 9,11-octadecadienoic acid and CLA-type compounds (e.g., 10-trans,12-cis-linoleic acid), adds a new dimension to the pharmacological potential of this plant. These lipids show activity via PPARγ and Nrf2 pathways, suggesting implications for metabolic and inflammatory disorders [142,143,144,145,146]. However, most of these findings are derived from mechanistic in vitro studies using non-purified fractions, which limits their direct pharmacological interpretation. Additional studies on bioavailability, metabolic stability, and formulation strategies are needed to assess their translational potential.

The discovery of previously undetected norisoprenoids, terpenoids, sulfur-containing heterocycles, and halogenated aromatics—such as indole-3-carboxaldehyde and 4,3′-difluoro-4′-methoxybiphenyl—expands the chemotaxonomic boundaries of the genus. These compounds, some with established biological relevance in other systems, such as AhR-mediated immunomodulation and antibacterial activity, provide leads for future drug discovery pipelines [10]. Their presence in *P. fruticosa*, particularly in lipophilic fractions, may support the species’ distinct biosynthetic repertoire within Araliaceae and merits comparative chemotaxonomic studies with *Panax* and *Aralia* species. Nevertheless, their low abundance and analytical sensitivity challenge isolation and characterization efforts, requiring the development of efficient enrichment protocols and high-resolution analytical tools.

Although numerous studies have cataloged the presence of triterpenoid saponins and flavonoids [3,4,7,8,17], their biosynthetic pathways in *P. fruticosa* remain largely uncharacterized. Comparative transcriptomic and metabolomic studies, especially in relation to *Panax* and *Aralia* species, could help clarify the evolution and regulation of secondary metabolite biosynthesis in *Polyscias* [30]. In our view, such studies would enable functional annotation of biosynthetic gene clusters and support metabolic engineering or biotechnological production strategies.

From a methodological perspective, the application of GC-MS has proven essential in revealing volatile and lipophilic components. Nevertheless, this technique has limitations in detecting thermolabile or highly polar molecules. For instance, compounds such as levoglucosan may represent pyrolytic artifacts rather than true endogenous metabolites. Future research would benefit from integrating LC-MS/MS, NMR-based metabolomics, and spatial metabolite mapping (e.g., MALDI imaging) to provide a more comprehensive profile. Moreover, method validation using authentic standards and isotopically labeled compounds is needed to confirm the endogenous nature of low-abundance features.

In conclusion, *P. fruticosa* should be considered a chemically rich and pharmacologically versatile species. Its documented pharmacological effects—including anti-inflammatory, antidiabetic, antioxidant, and cytoprotective activities—can now be linked to a broader spectrum of structurally diverse metabolites.

The underutilization of its potential is primarily due to the limited integration of biosynthetic, pharmacokinetic, and clinical investigations. A systematic research agenda focusing on bioactivity-guided isolation, in vivo efficacy, and molecular mechanisms will be instrumental in unlocking its full therapeutic potential.

We also emphasize the need for chemotaxonomic and phylogenetic studies to better position *P. fruticosa* within the metabolic diversity of Araliaceae and to identify unique metabolite signatures that may guide drug discovery and standardization efforts.

## 6. Pharmacological Properties of *Polyscias fruticosa*: An Overview of Current Evidence

*Polyscias fruticosa* has been extensively used in traditional medicine across Southeast Asia for the management of inflammatory disorders, respiratory diseases, metabolic dysfunctions, and stress-related conditions. The pharmacological interest in this species stems not only from its diverse ethnomedicinal applications and the growing body of experimental evidence supporting its therapeutic potential. Notably, in vitro and in vivo studies have demonstrated anti-inflammatory, antiasthmatic, antidiabetic, antioxidant, antimicrobial, and neuroprotective activities [4,10,15,16,17,20,21].

A comprehensive characterization of the phytochemical constituents of *P. fruticosa*—including triterpenoid saponins, flavonoids, sterols, polyacetylenes, phenolic acids, and various volatile and lipophilic compounds—has been provided in Section 5: Chemical Composition and Structural Diversity of Bioactive Compounds in *Polyscias fruticosa*. Accordingly, the present section focuses exclusively on summarizing the current pharmacological findings and elucidating the underlying mechanisms of action where available.

This section aims to synthesize current experimental findings related to the biological activities of *P. fruticosa*, with particular emphasis on pharmacodynamic mechanisms and disease-relevant applications.

Although the current body of pharmacological evidence is promising, it remains fragmented and largely limited to preclinical studies. In our opinion, a critical gap persists in the form of well-designed in vivo studies and clinical trials that would validate the therapeutic relevance of *P. fruticosa*. Furthermore, few studies have directly linked specific compounds to distinct bioactivities using bioassay-guided isolation or mechanistic validation. Addressing these limitations will require interdisciplinary approaches combining phytochemistry, pharmacology, and systems biology to elucidate compound–target interactions and therapeutic pathways.

### 6.1. Bronchial Asthma—Clinical Background and Treatment Challenges

Bronchial asthma is a chronic inflammatory disorder of the airways characterized by variable clinical symptoms and reversible airflow limitation. It affects both children and adults, with global prevalence steadily rising. According to the World Health Organization, more than 300 million people are affected worldwide, with projections exceeding 400 million by 2025 [153].

The pathophysiology of asthma is complex, involving genetic predispositions, environmental exposures, and dysregulated immune responses. A hallmark feature is eosinophilic inflammation leading to hyperresponsiveness, obstruction, and structural airway remodeling. T-helper type 2 (Th2) lymphocytes play a central role by secreting IL-4, IL-5, and IL-13, which promote IgE production, eosinophil recruitment, and mediator release from mast cells [154].

Chronic inflammation also induces basement membrane thickening, smooth muscle hypertrophy, and mucus hypersecretion.

Pharmacologic management of asthma typically involves inhaled corticosteroids (ICS), β2-adrenergic agonists, leukotriene receptor antagonists, anticholinergic agents, and targeted biologic therapies such as monoclonal antibodies against IL-5, IL-4Rα, and IgE. However, many patients continue to experience exacerbations, and long-term drug use may cause adverse effects that reduce adherence and efficacy [153].

These limitations have fueled interest in complementary plant-based therapies with anti-inflammatory, antioxidant, and immunomodulatory potential. Among these is *P. fruticosa*, a member of the Araliaceae family that is traditionally used in the treatment of inflammatory conditions, respiratory ailments, and as a general tonic [4,155]. Recent studies suggest that *P. fruticosa* extracts can modulate airway inflammation, oxidative stress, bronchial tone, and mucus secretion, supporting its potential as a phytotherapeutic agent in asthma [10,56].

#### 6.1.1. Antiasthmatic Potential of *Polyscias fruticosa*: Pharmacological and Mechanistic Insights

The ovalbumin (OVA)-induced asthma model is a widely accepted preclinical approach for evaluating immunopathological features and therapeutic responses in allergic asthma. It reproduces hallmark features of human asthma, such as eosinophilic airway inflammation, bronchial hyperresponsiveness, increased IgE levels, and a Th2-skewed cytokine profile.

In a study by Koffuor et al. [56], guinea pigs were sensitized and challenged with OVA and subsequently treated orally with an ethanolic leaf extract of *P. fruticosa* (PFE) at doses of 100, 250, and 500 mg·kg^−1^ for 21 consecutive days. Therapeutic efficacy was assessed via BALF eosinophil counts, serum CRP, ESR, and histopathological analysis of lung tissue.

Administration of PFE at (500 mg·kg^−1^) resulted in a 56.3% reduction in eosinophils, a 43% decrease in CRP, and notable histological improvements. The treated animals showed reduced peribronchial infiltration and preserved bronchial epithelium. These effects were comparable to those of prednisolone (2 mg·kg^−1^), underscoring the extract’s anti-inflammatory potency.

Although cytokine levels were not directly measured, the results suggest Th2 suppression-possibly via reduced IL-4, IL-5, and IL-13 activity. Furthermore, hematological assessments and histological analysis of major organs revealed no signs of systemic toxicity, indicating the safety of prolonged PFE administration.

These findings confirm the antiasthmatic potential of *P. fruticosa*, particularly in reducing airway inflammation and systemic inflammatory markers, without inducing toxicity, supporting its development as a phytotherapeutic for allergic asthma [56]. These results confirm the antiasthmatic activity of *P. fruticosa* in vivo; however, the lack of cytokine data limits mechanistic understanding. Further studies should clarify immunomodulatory pathways and identify active constituents.

#### 6.1.2. Mucolytic and Antitussive Activity

Effective control of mucus hypersecretion and cough is critical in the management of symptomatic asthma, particularly in mucus-dominant phenotypes. In a study conducted by Koffuor et al. [18], two complementary animal models were employed to assess the mucolytic and antitussive effects of *P. fruticosa*: (1) ammonium chloride (NH_4_Cl)-induced bronchial secretion in ICR mice and (2) citric-acid-induced cough in guinea pigs. An aqueous root extract of *P. fruticosa* (PFE) was administered orally at doses of 100, 200, and 400 mg·kg^−1^ body weight.

In the NH_4_Cl-induced mucus model, semi-quantitative evaluation using phenol red dye revealed a significant reduction in bronchial secretion. PFE at 400 mg·kg^−1^ reduced mucus volume by 49.7% versus control, with efficacy comparable to bromhexine, a reference mucolytic agent. The dose-dependent nature of this response indicates a consistent pharmacodynamic effect of the extract.

In the citric-acid-induced cough model, PFE significantly reduced the number of cough episodes by 67% and prolonged the latency to first cough by 82%, compared to the vehicle-treated group. These results indicate both suppression of chemically induced cough and possible modulation of airway sensory responsiveness. The antitussive effect was comparable to that of dihydrocodeine, a centrally acting reference drug.

Biochemical safety assessments, including serum levels of alanine aminotransferase (ALT), aspartate aminotransferase (AST), creatinine, and urea, showed no significant deviations from normal values, confirming the absence of systemic toxicity following subacute oral administration of PFE at doses up to 400 mg·kg^−1^.

Triterpenoid saponins and glycosides may underlie the mucolytic effect, potentially by promoting mucociliary clearance and epithelial hydration. Although the precise mechanism of the antitussive action remains unclear, the observed effects suggest peripheral modulation of the cough reflex, possibly through desensitization of vagal afferents or through the regulation of TRPV1 and TRPA1 ion channels, both of which are involved in mediating airway sensory signaling [18].

In our opinion, these findings support the therapeutic relevance of *Polyscias fruticosa* in managing airway symptoms such as mucus hypersecretion and cough. Nonetheless, further studies are needed to elucidate its underlying mechanisms and confirm the observed effects in clinical settings.

#### 6.1.3. Bronchodilatory and Antihistaminic Effects

The ability to induce bronchial smooth muscle relaxation and counteract the effects of bronchoconstrictors such as histamine and acetylcholine is central to the pharmacological management of asthma. In a study by Koffuor et al. [15], the bronchodilatory effects of *P. fruticosa* extract (PFE) was evaluated in guinea pigs subjected to bronchospasm induced by inhalation of histamine (5.8 × 10^−2^ mg·mL^−1^) and acetylcholine (2.0 × 10^−2^ mg·mL^−1^). Following provocation, animals received oral doses of PFE at 100, 250, and 500 mg·kg^−1^, and responses were compared to those of reference drugs: mepyramine (an H_1_ receptor antagonist) and atropine (a muscarinic M_3_ receptor antagonist).

PFE significantly prolonged the time to onset of pre-convulsive dyspnea (PCD) in a dose-dependent manner, with increases ranging from 70% to 110%. It also reduced recovery time by 44–63% compared to control, confirming effective bronchodilation. These bronchodilatory effects were corroborated by in vitro experiments using isolated guinea pig ileum, in which PFE attenuated contractile responses to exogenously applied histamine and acetylcholine, indicating potential antagonism at H_1_ and M_3_ receptors [15].

These effects may be mediated by flavonoids, sesquiterpenes, and alkaloids that modulate G protein-coupled receptors regulating airway tone. Additionally, PFE may stabilize mast cells, reducing the release of bronchoconstrictors such as histamine, prostaglandins, and leukotrienes.

No adverse effects were reported at oral doses up to 500 mg·kg^−1^, supporting the safety profile of the extract under subacute exposure conditions [15,18].

Collectively, the data support the bronchodilatory and antihistaminic potential of *P. fruticosa*, highlighting its relevance as a safe phytotherapeutic option for obstructive airway conditions including asthma and COPD.

In our opinion, these findings highlight the potential of *P. fruticosa* as a dual-acting bronchodilator and antihistaminic agent. However, further mechanistic studies and clinical validation are warranted to confirm its role in airway pharmacotherapy.

#### 6.1.4. Molecular Mechanisms: Anti-Inflammatory and Antioxidant Pathways

Contemporary pharmacological research emphasizes the need to elucidate molecular mechanisms in order to identify therapeutic targets of plant-derived compounds. In this regard, Rarison et al. [10] employed an integrative approach combining network pharmacology, molecular docking, and in vitro assays using LPS-stimulated RAW 264.7 macrophages to evaluate the bioactivity of lipophilic extracts from *P. fruticosa*. The extract, tested at concentrations of 12.5, 25, and 50 µg·mL^−1^, significantly inhibited nitric oxide (NO) production without inducing cytotoxicity, as confirmed by MTT assay. These effects were consistent with in silico predictions, indicating interactions with inflammation-related targets such as NF-κB, MAPK1, and PTGS2. A total of 71 phytoconstituents were identified in the extract, including sesquiterpenes (e.g., α-humulene), phytosterols (e.g., β-sitosterol), flavonoids, and phenolic acids. Molecular docking revealed strong binding to proteins involved in inflammatory and oxidative stress pathways, such as PTGS2 (COX-2), KEAP1, NFKB1, PRKCD, and TLR4. These findings suggest a dual modulatory potential—both anti-inflammatory and antioxidant.

In vitro assays further demonstrated that the extract significantly reduced the expression of pro-inflammatory cytokines TNF-α, IL-1β, and IL-6 in LPS-activated RAW 264.7 cells. ELISA confirmed cytokine suppression, while RT-qPCR and Western blotting showed downregulation at both mRNA and protein levels.

Importantly, the extract also upregulated the expression of antioxidant enzymes, including heme oxygenase-1 (HO-1) and catalase, which are under transcriptional control of the nuclear factor erythroid 2-related factor 2 (Nrf2). This indicates concurrent activation of the Nrf2-ARE pathway and inhibition of the TLR4-NF-κB inflammatory axis, contributing to a broad-spectrum protective mechanism [10].

Given the interplay between oxidative damage and chronic inflammation in asthma pathogenesis, the multimodal activity of *P. fruticosa* is of particular therapeutic interest.

By simultaneously suppressing pro-inflammatory signaling and enhancing cytoprotective defenses, *P. fruticosa* may aid both symptom management and long-term disease control in asthma, where oxidative stress and inflammation are synergistic contributors.

In our opinion, the ability of *P. fruticosa* extracts to simultaneously modulate key inflammatory and antioxidant pathways provides a strong rationale for further mechanistic and translational studies. Future work should focus on compound-specific activity and in vivo confirmation to validate therapeutic relevance.

### 6.2. Antidiabetic Activity of Polyscias fruticosa

#### 6.2.1. Type 2 Diabetes and the Need for New Therapies

Diabetes mellitus (DM) is a chronic, progressive metabolic disorder and one of the most urgent global health challenges of the 21st century. Data from the World Health Organization indicated a global prevalence of 2.8% in 2000, expected to reach 5.4% by 2025 [156]. More recent estimates suggest that by 2045, nearly 700 million individuals—approximately 10% of the global adult population—will be affected [157]. The burden is especially high in low- and middle-income countries, where urbanization, dietary changes, and limited healthcare access accelerate incidence.

Type 2 diabetes mellitus (T2DM), which represents over 90% of DM cases, is primarily characterized by insulin resistance and a relative deficiency in insulin secretion. Chronic hyperglycemia, the hallmark of T2DM, is a major driver of both microvascular complications (e.g., nephropathy, retinopathy, neuropathy) and macrovascular diseases (e.g., coronary artery disease, stroke, peripheral arterial disease) [158].

Pharmacological management of T2DM typically involves oral antihyperglycemic agents such as metformin, sulfonylureas, dipeptidyl peptidase-4 (DPP-4) inhibitors, sodium–glucose co-transporter 2 (SGLT2) inhibitors, glucagon-like peptide-1 (GLP-1) receptor agonists, and exogenous insulin therapy. Although effective for glycemic control, these drugs are associated with reduced long-term efficacy, adverse effects (e.g., hypoglycemia, gastrointestinal intolerance), and limited adherence [156,158]. In resource-limited settings, high cost and restricted access further hinder disease management [157].

These limitations have driven interest in plant-based therapies as adjuncts or alternatives. Numerous phytochemicals—including flavonoids, triterpenoids (notably oleanolic acid), saponins, phenolic acids, and coumarins—have been shown to exert antidiabetic effects through diverse mechanisms. These include insulin-mimetic activity, stimulation of pancreatic β-cell insulin secretion, inhibition of carbohydrate-digesting enzymes (e.g., α-glucosidase, α-amylase), enhancement of peripheral glucose uptake, and modulation of inflammation and oxidative stress [158].

Several species within the Araliaceae family, including *P. fruticosa*, have been traditionally used in the management of diabetes. These plants are rich in triterpenoid saponins, oleanolic acid derivatives, and a wide array of polyphenols, all of which have demonstrated antidiabetic properties in preclinical studies.

Thus, *P. fruticosa* is considered a promising candidate for developing affordable, multi-targeted phytotherapeutics to address the complex pathophysiology of T2DM.

#### 6.2.2. Mechanisms of Action of Identified Compounds

*Polyscias fruticosa*, a medicinal plant widely utilized in traditional Southeast Asian practices, is a rich source of bioactive triterpenoid saponins primarily derived from oleanolic acid. Among these, the most extensively studied antidiabetic compound is 3-*O*-[β-D-glucopyranosyl-(1→4)-β-D-glucuronopyranosyl] oleanolic acid 28-*O*-β-D-glucopyranosyl ester, commonly referred to as PFS [57].

In vitro enzymatic assays have demonstrated that PFS exhibits potent inhibitory activity against two key carbohydrate-hydrolyzing enzymes: α-amylase and α-glucosidase. Kinetic studies showed mixed noncompetitive inhibition of α-amylase and noncompetitive inhibition of α-glucosidase [57]. These dual inhibitory effects delay the enzymatic breakdown of dietary carbohydrates in the small intestine, thereby reducing glucose absorption and attenuating postprandial glycemic elevation.

The antidiabetic effect of PFS has also been confirmed in vivo. In a diet-induced hyperglycemic mouse model, oral administration of PFS at 100 mg·kg^−1^ significantly reduced blood glucose levels in a sucrose tolerance test, as evidenced by a lower area under the curve (AUC) [57].

Together, these findings support the multitargeted antidiabetic mechanism of PFS, combining inhibition of key digestive enzymes with effective reduction in glycemic response in vivo.

In our opinion, the complexity of T2DM pathophysiology justifies the exploration of multi-targeted phytotherapeutics, such as *P. fruticosa*. Its rich phytochemical profile warrants further investigation in translational and clinical contexts.

#### 6.2.3. In Vivo Evidence of Antidiabetic Activity

In vivo studies have substantiated the hypoglycemic properties of PFS isolated from *P. fruticosa*. In BALB/c mice subjected to a high-sucrose diet, oral administration of PFS (100 mg·kg^−1^ body weight) significantly reduced postprandial blood glucose levels following a sucrose challenge. This effect was reflected by a significant reduction in the area under the glucose curve (AUC), consistent with delayed digestion and absorption of carbohydrates due to enzymatic inhibition [57].

Moreover, Tran et al. [14] reported that co-administration of low-dose PFS with acarbose enhanced the inhibition of α-amylase, producing a synergistic effect superior to either agent alone. This suggests a potential adjuvant role of PFS, enabling lower doses of conventional antidiabetic drugs and minimizing side effects.

In our opinion, the observed in vivo effects of PFS demonstrate promising hypoglycemic activity via enzymatic inhibition and synergism with standard drugs. However, further studies are needed to elucidate the bioavailability, pharmacokinetics, and long-term safety of PFS. In addition, the specific saponins responsible for this effect remain to be identified and characterized in detail. These insights would enhance the translational potential of *P. fruticosa*-based formulations in T2DM management.

#### 6.2.4. Structure–Activity Relationship and Glycosidic Considerations

Structure–activity relationship (SAR) studies of oleanolic saponins have shown that the position and type of sugar substitution strongly influence antidiabetic activity. Guo et al. [159] demonstrated that 28-O-monoglycosides display higher α-glucosidase inhibition than 3-O-substituted analogs, which may be sterically hindered. Additionally, the presence of a terminal α-L-rhamnose enhances potency, likely via improved enzyme binding.

Substitution at C-28 favors interaction with hydrophobic pockets of digestive enzymes, whereas large glycosidic chains at C-3 can hinder access to catalytic sites. [159].

In our opinion, these SAR insights are particularly relevant to *P. fruticosa*, which contains oleanolic acid-type saponins with diverse sugar moieties. However, the glycosylation patterns of PFS remain incompletely characterized, and structure-guided isolation of specific glycosides may help identify the most potent antidiabetic constituents. Future SAR studies on purified saponins from *P. fruticosa* are warranted to optimize their therapeutic efficacy.

#### 6.2.5. Molecular Mechanisms in Diabetes: Anti-Inflammatory and Antioxidant Pathways

Increasing in vitro and in vivo data support the multimodal effects of *P. fruticosa* extracts in T2DM (T2DM). Le et al. [160] reported that co-administration of *P. fruticosa* and *Morus alba* alleviated diabetic complications, including neuropathy and retinopathy, indicating synergistic metabolic and vascular protection.

Among identified compounds, PFS remains the lead candidate due to its enzymatic inhibition profile, favorable tolerability, and the potential to optimize glycosidic moieties for enhanced bioactivity.

Although glycosylation increases aqueous solubility, it may compromise absorption by lowering lipophilicity and membrane permeability. Therefore, rational modification of glycosidic residues is a promising strategy to fine-tune pharmacokinetic and pharmacodynamic profiles of oleanolic saponins in antidiabetic therapy. In our opinion, targeted structural modifications of glycosidic moieties—particularly at C-3 and C-28 positions—could significantly improve the therapeutic performance of *P. fruticosa* saponins. Future studies should explore structure–absorption relationships and evaluate bioavailability using advanced in vivo models.

#### 6.2.6. Pharmacokinetics and Bioavailability of PFS

Chronic low-grade inflammation and oxidative stress are major drivers of insulin resistance and pancreatic β-cell dysfunction in T2DM. Pro-inflammatory cytokines such as TNF-α, IL-1β, and IL-6 impair insulin signaling via downregulation of IRS-1 and inhibition of the PI3K/AKT pathway [57].

PFS, the oleanolic-acid-based saponin from *P. fruticosa*, exhibits anti-inflammatory effects by suppressing the NF-κB pathway, leading to decreased transcription of TNF-α, IL-1β, and IL-6 [57]. Concurrently, PFS activates the Nrf2-ARE signaling cascade, promoting the expression of antioxidant enzymes such as HO-1, catalase, and superoxide dismutase (SOD), thereby enhancing cellular defense against oxidative stress.

Through simultaneous modulation of inflammatory and redox-sensitive pathways, PFS demonstrates pleiotropic antidiabetic action, supporting its potential as a disease-modifying phytochemical in the management of T2DM.

In our opinion, the ability of PFS to simultaneously attenuate inflammatory signaling and enhance antioxidant defenses provides a mechanistic rationale for its use as a multi-target antidiabetic agent. Further studies are needed to confirm these effects in vivo and to assess their impact on insulin sensitivity and β-cell function.

#### 6.2.7. Safety and Toxicity of *Polyscias fruticosa* Preparations

Preclinical safety data indicate a favorable toxicological profile for *P. fruticosa* extracts. In vitro cytotoxicity assays using mammalian cell lines showed no cytotoxic effects of aqueous and ethanolic extracts from leaves or roots, even at high concentrations [158].

Likewise, oral administration of PFS up to 100 mg·kg^−1^ in murine models produced no signs of hepatotoxicity, nephrotoxicity, or histological damage to major organs. Behavioral and metabolic parameters remained stable, supporting the extract’s safety under subacute exposure conditions [158].

Traditional uses of *P. fruticosa* as food and herbal medicine across Southeast Asia, e.g., in teas, soups, and salads, offers ethnobotanical evidence of its tolerability. Its dietary application, without documented adverse events, further reinforces its safety [158].

However, the amphiphilic structure of triterpenoid saponins such as PFS may interact with erythrocyte membranes, posing a theoretical risk of hemolysis at supraphysiological concentrations [57].

Although no hemolytic effects have been observed at therapeutic doses, further studies are warranted to evaluate chronic toxicity, reproductive safety, and genotoxicity.

For clinical application, standardized formulations of *P. fruticosa* are essential, including detailed profiling of active saponins to ensure consistency and regulatory compliance.

In our opinion, further toxicological evaluation—particularly on chronic exposure, reproductive safety, and genotoxicity—is crucial for clinical translation. Future studies should also address herb–drug interactions and human metabolism of key saponins.

#### 6.2.8. Comparison with Other Antidiabetic Plants

Among medicinal plants investigated for their antidiabetic activity, *P. fruticosa* emerges as a particularly promising candidate due to its multifaceted pharmacological profile. Its principal bioactive constituent—PFS, chemically defined as 3-*O*-[β-D-glucopyranosyl-(1→4)-β-D-glucuronopyranosyl] oleanolic acid 28-*O*-β-D-glucopyranosyl ester—exhibits potent dual inhibition of both α-amylase and α-glucosidase, key enzymes involved in postprandial glucose regulation [57]. This enzymatic inhibition is further complemented by the extract’s anti-inflammatory properties, mediated via suppression of the NF-κB signaling cascade, and its stimulation of the Nrf2-dependent antioxidant response pathway [160].

In comparison, *Morus alba* (white mulberry) exerts its hypoglycemic effect primarily through 1-deoxynojirimycin (DNJ), a selective α-glucosidase inhibitor with limited or no activity against α-amylase. As a result, its ability to attenuate postprandial hyperglycemia may be less pronounced than that of multitarget agents such as PFS [160]. Moreover, *M. alba* lacks strong evidence of anti-inflammatory or antioxidant effects, which limits its utility in addressing the complex pathophysiology of T2DM.

*Momordica charantia* (bitter melon) contains insulin-mimetic compounds that promote glucose uptake and enhance glycemic control; however, marked variability in phytochemical composition among cultivars and geographic origins has been shown to affect the reproducibility of its hypoglycemic effects [156]. Similarly, *Panax notoginseng* exerts antidiabetic effects mainly through ginsenosides that improve insulin sensitivity and β-cell function, yet the overall magnitude and consistency of these effects remain variable, and the cost and standardization of ginsenoside-rich extracts present additional challenges [161].

A noteworthy comparison can be drawn with *Aralia elata*, a close relative of *P. fruticosa* within the Araliaceae family. This species contains oleanolic acid and triterpenoid saponins such as araloside A, which have demonstrated inhibition of aldose reductase, a key enzyme involved in diabetic complications, and enhancement of glucose homeostasis [157]. This suggests a conserved triterpenoid-based antidiabetic mechanism within the Araliaceae family.

The multitarget pharmacodynamic profile of *P. fruticose*—encompassing digestive enzyme inhibition, modulation of inflammatory signaling, and activation of redox-regulatory pathways—distinguishes it from other botanicals with more limited mechanisms of action. In addition to its low toxicity and traditional dietary use in Southeast Asia, these properties support its potential as a safe, culturally accepted, and biologically effective phytotherapeutic agent.

Unlike single-mechanism herbs such as *M. alba*, *P. fruticosa* may yield additive or synergistic effects when co-administered with conventional antidiabetic agents. For instance, co-administration with acarbose has demonstrated additive inhibitory effects on carbohydrate-digesting enzymes, supporting its role in combinatorial phytotherapy [14].

Further comparative studies in diabetic animal models and human trials are warranted to confirm the clinical relevance and superiority of *P. fruticosa* in managing type 2 diabetes mellitus.

In our opinion, the unique multitarget action of *P. fruticosa* positions it as a more comprehensive phytotherapeutic candidate compared to single-mechanism botanicals. Its synergy with standard drugs further increases its translational relevance in modern diabetes management.

### 6.3. Potential of Polyscias fruticosa in Neurodegenerative Diseases

Neurodegenerative diseases such as Parkinson’s disease (PD) and Alzheimer’s disease (AD) are characterized by progressive neuronal loss and currently lack effective disease-modifying therapies. Although pharmacological treatments such as levodopa and dopamine agonists offer symptomatic relief, their prolonged use is associated with significant side effects, including neurotoxicity, motor fluctuations, and dyskinesias [4,20,21,162]. These limitations have stimulated growing interest in medicinal plants such as *P. fruticosa* as potential alternative or adjunct therapies.

Multiple studies have shown that phytochemicals—particularly polyphenols, flavonoids, alkaloids, and saponins—can modulate oxidative stress, mitochondrial dysfunction, and neuroinflammation, which are central mechanisms underlying neurodegeneration [21,162]. *P. fruticosa*, a rich source of these bioactive compounds, has demonstrated antioxidant, anti-inflammatory, and neurotrophic properties relevant to neurodegenerative pathologies [20,21].

#### 6.3.1. Molecular Mechanisms and Comparative Neuroprotective Potential of *Polyscias fruticosa*

The neuroprotective activity of *P. fruticosa* has been demonstrated in both in vivo and in vitro models. In a murine model of Parkinson’s disease induced by MPTP, Ly et al. [20] reported that oral administration of ethanolic leaf extract significantly restored dopaminergic neuronal density in the substantia nigra and increased striatal dopamine levels. These effects were associated with activation of the AKT/CREB/BDNF signaling cascade, which supports neuronal survival and synaptic plasticity. Concurrently, the extract suppressed NF-κB activation and reduced the expression of pro-inflammatory cytokines, including IL-1β, IL-6, and TNF-α, as shown by Western blot and immunohistochemical analyses [20,163].

Supporting in vitro evidence was provided by Selvaraj et al. [21], who treated murine HT22 hippocampal neurons with a 75% ethanol extract of *P. fruticosa* roots (EEPF). At 50 µg·mL^−1^, EEPF markedly reduced glutamate-induced oxidative stress (assessed by DCF-DA fluorescence) and intracellular Ca^2+^ influx (measured with Fluo-3-AM). The extract also inhibited apoptosis by preventing AIF nuclear translocation and restored AKT and CREB phosphorylation and BDNF expression. Western blot analysis revealed modulation of MAPK phosphorylation and normalization of the Bax/Bcl-2 ratio, indicating mitochondrial pathway engagement.

Taken together, available evidence suggests that the neuroprotective effects of *P. fruticosa* are mediated by suppression of pro-inflammatory cytokines, ROS neutralization, mitochondrial stabilization, and activation of neurotrophic signaling pathways [8]. These multimodal mechanisms are relevant to the pathogenesis of disorders such as Parkinson’s and Alzheimer’s disease [20,21]. A meaningful comparison can be made with *Panax ginseng*, whose neuroprotective effects are mainly linked to ginsenosides like Rg3 acting via the PI3K/AKT pathway [20,164]. In contrast, *P. fruticosa* contains phenolic acids with potent antioxidant capacity (e.g., dehydroferulic acid, protocatechuic acid) and uniquely protects against glutamate excitotoxicity [21].

In our view, the broader mechanism of action of *P. fruticosa*, combining anti-inflammatory, antioxidant, and mitochondrial-stabilizing effects, may confer advantages over classical neuroprotective plants. Its distinct phytochemical profile supports further investigation as an alternative or complementary agent in neurodegenerative disease therapy.

#### 6.3.2. Safety and Therapeutic Relevance

Toxicological assessments confirm the favorable safety profile of *P. fruticosa* extracts in preclinical models. Ly et al. [20] showed that repeated oral administration of ethanolic leaf extract at 100 mg·kg^−1^ for 14 days in MPTP-treated mice did not induce histopathological alterations in major organs. Hematological and biochemical parameters remained within normal ranges, suggesting systemic tolerability. This contrasts with synthetic dopaminergic agents that often induce hepatotoxicity and behavioral abnormalities upon chronic administration [20].

Selvaraj et al. [21] further demonstrated that co-treatment of HT22 neurons with *P. fruticosa* extract (50 µg·mL^−1^) and subtoxic doses of glutamate significantly enhanced cell viability compared to monotherapies, indicating potential synergism and a dose-sparing effect.

The multitarget pharmacological profile of *P. fruticosa*—encompassing antioxidant, anti-inflammatory, mitochondrial protective, and neurotrophic mechanisms—together with its low toxicity, supports its potential use as an adjunct neuroprotective agent. Further studies in translational animal models and clinical settings are warranted to validate its therapeutic utility in neurodegenerative disorders.

### 6.4. Antibacterial Properties of Polyscias fruticosa

The rapid emergence of antibiotic-resistant bacterial strains poses a serious threat to global health. The World Health Organization has identified antimicrobial resistance as one of the major public health challenges of the 21st century, with projections estimating up to 10 million annual deaths attributable to drug-resistant infections by 2050 [163]. The stagnation in the development of new antibiotics has intensified interest in alternative antimicrobial strategies, particularly those based on medicinal plants [165]. Medicinal plant-derived phytochemicals exhibit multitarget antibacterial activity and are increasingly explored as complementary agents against resistant pathogens. Plant-derived extracts constitute a rich reservoir of bioactive compounds capable of exerting broad-spectrum antibacterial effects through multiple mechanisms, such as membrane disruption, inhibition of macromolecular synthesis, and suppression of biofilm formation [166].

Within this context, *P. fruticosa*, a member of the Araliaceae family, has attracted interest due to its diverse phytochemical composition and experimentally validated antibacterial potential [16,17,94].

#### 6.4.1. Phytochemical Composition and Antibacterial Potential

*Polyscias fruticosa* is known to contain a broad array of bioactive metabolites, including triterpenoid saponins (e.g., ladyginoside A, chikusetsusaponin IVa, polysciosides A–K), flavonoids (e.g., quercetin), phenolic acids (e.g., gallic acid), alkaloids, tannins, and polyacetylenes such as falcarinol and heptadeca-1,8(E)-diene-4,6-diyne-3,10-diol [10,94]. Among these, polyacetylenes have demonstrated particularly strong antibacterial activity, frequently surpassing that of saponin-rich fractions [10]. Comparative phytochemical analysis showed that ether-soluble fractions enriched in polyacetylenes (EPA) exhibited greater antibacterial potency than n-butanol fractions (NBHS) containing primarily triterpenoid saponins [10].

These findings suggest that polyacetylenes are likely the primary contributors to the antibacterial effects of *P. fruticosa*.

#### 6.4.2. Comparative Antibacterial Efficacy

The antibacterial activity of *P. fruticosa* compares favorably with that of other medicinal plants. In the study by Titova et al. [17], extracts derived from *P. fruticosa* suspension cultures grown in bubble-type bioreactors exhibited minimum inhibitory concentration (MIC) values ranging from 250 to 2000 µg·mL^−1^, markedly lower than the MICs of 4000 µg·mL^−1^ reported for greenhouse-derived leaf extracts. These values also outperform those of *Thymus vulgaris* and *Syzygium aromaticum*, which exhibited MICs of approximately 3150 µg·mL^−1^ against *Bacillus cereus*, and *Rosmarinus officinalis*, which required >1000 µg·mL^−1^ to inhibit *E. coli* and *Staphylococcus aureus* [166].

In contrast, *Panax notoginseng*, a related Araliaceae species, displayed more limited antibacterial activity, primarily targeting *Streptococcus pyogenes*, with diminished efficacy against Gram-negative bacteria [167,168].

These comparisons underscore the broad-spectrum potential of *P. fruticosa*, particularly due to its polyacetylene-rich composition and favorable safety profile.

#### 6.4.3. Evidence of Antibacterial Activity

Several studies have confirmed the antibacterial efficacy of *P. fruticosa* against clinically relevant pathogens, including *Staphylococcus aureus* (including methicillin-resistant *S. aureus*, MRSA), *E. coli*, *Pseudomonas aeruginosa*, and *Salmonella typhi* [16,17,94]. In particular, Titova et al. [17] reported superior activity of extracts obtained from in vitro suspension cultures, which produced significantly lower MICs than those derived from field-grown material.

Polyacetylene-rich fractions were consistently more effective than triterpenoid saponin fractions [10], suggesting that lipophilic compounds may better penetrate bacterial membranes or disrupt vital functions. Importantly, these extracts demonstrated no acute toxicity in murine models [10], supporting their potential safety as natural antimicrobial agents.

#### 6.4.4. Mechanisms of Antibacterial Action

The antibacterial effects of *P. fruticosa* are believed to result from multiple synergistic mechanisms:

- Disruption of bacterial membrane integrity—both saponins and polyacetylenes can increase membrane permeability, leading to osmotic imbalance and cell lysis [165,166,167,168].
- Inhibition of protein and peptidoglycan biosynthesis—certain constituents may bind to ribosomal subunits or enzymes that are critical for bacterial cell wall synthesis, disrupting growth and replication [17].
- Alteration of membrane potential and intracellular pH—some extracts interfere with bacterial membrane electrochemical gradients, leading to acidification and metabolic dysfunction [165].
- Inhibition of biofilm formation—compounds such as falcarinol and flavonoids impair bacterial adhesion and prevent the establishment of protective biofilm matrices [17,94].

These multitarget mechanisms may enhance effectiveness against antibiotic-resistant strains and reduce the likelihood of resistance development. Further mechanistic studies using molecular and omics approaches are warranted to fully elucidate the antimicrobial pathways involved.

### 6.5. Phytochemical Significance of Polyscias fruticosa in Green Synthesis of Anticancer Nanoparticles

The use of plant-derived bioactive compounds in nanomedicine is gaining traction as a strategy to enhance the efficacy, selectivity, and safety of anticancer agents. *Polyscias fruticosa*, traditionally recognized for its anti-inflammatory and immunomodulatory properties, has recently gained attention as a valuable resource for the green synthesis of metal-based nanoparticles due to its content of triterpenoid saponins, polyphenols, and flavonoids [3,4,8,10,11,13].

These phytoconstituents play a dual role: (i) acting as natural reducing agents facilitating the conversion of metal ions into nanoparticles, and (ii) stabilizing the resulting nanostructures by improving colloidal stability and bioavailability [169,170].

Such dual functionality supports its use in environmentally sustainable nanotechnological approaches, particularly in biomedical applications such as cancer therapy.

#### 6.5.1. Biosynthesis and Cytotoxicity of Nanoparticles

Recent studies have confirmed the feasibility of green synthesis of silver (AgNPs) and copper (CuNPs) nanoparticles using aqueous or ethanolic extracts from *P. fruticosa* leaves and roots [169,170]. These biosynthesized nanoparticles exhibit spherical morphology (50–120 nm), characteristic UV-Vis plasmon resonance peaks, and negative zeta potentials (−25 to −30 mV), indicating stability and uniformity.

Cytotoxicity assays demonstrated that Pfr-AgNPs reduced the viability of A549 human lung carcinoma cells by 47.3% at 100 µg·mL^−1^, while Pfr-CuNPs inhibited neuroblastoma cell proliferation by 55.5% at 125 µg·mL^−1^ [169,170]. Mechanistically, cytotoxic effects are associated with ROS generation, mitochondrial dysfunction, membrane depolarization, and caspase-mediated apoptosis.

Notably, brine shrimp lethality assays confirmed the low toxicity of the crude extract (LC_50_ > 1000 µg·mL^−1^), whereas the nanoparticles exhibited moderate toxicity (LC_50_ = 524 µg·mL^−1^), suggesting selective cytotoxicity toward cancer cells.

#### 6.5.2. Mechanistic Insights and Therapeutic Prospects

Current evidence indicates that the cytotoxic effects of *P. fruticosa*-derived nanoparticles are mainly due to the release of Ag^+^ or Cu^+^ ions, which induce intracellular oxidative stress, apoptosis pathways, and inhibition of cellular proliferation [171]. Additionally, phytochemical surface coatings composed of saponins and flavonoids may facilitate cellular uptake and improve intracellular delivery.

Triterpenoid saponins present in the extract are hypothesized to influence multiple molecular targets, including MAPK signaling, topoisomerase inhibition, and regulation of cell cycle genes and metastasis-related pathways, offering a multimodal anticancer mechanism.

However, further studies are required to evaluate the in vivo efficacy, biodistribution, and long-term safety of these nanoparticles, as the current evidence is limited to in vitro models.

### 6.6. Reproductive Effects of Polyscias fruticosa

Medicinal plants have played a foundational role in the management of reproductive disorders within various traditional medical systems. Their phytochemical complexity, including phytoestrogens, alkaloids, flavonoids, and saponins, enables modulation of endocrine pathways and offers potential therapeutic alternatives to conventional hormone-based interventions [172]. Botanical species such as *Trifolium pratense* (red clover), *Withania somnifera* (ashwagandha), and *Panax ginseng* have demonstrated efficacy in regulating folliculogenesis, improving hormonal balance, and enhancing fertility parameters in both females and males [172].

In this context, *P. fruticosa*, widely recognized for its adaptogenic, anti-inflammatory, and immunomodulatory properties, has recently attracted attention for its potential reproductive effects. Emerging preclinical data suggest that its extracts modulate both ovarian and testicular function through phytohormonal and antioxidant pathways.

#### 6.6.1. Female Reproductive Effects

Boye et al. [173] investigated the influence of *P. fruticosa* leaf extract (PFE) on ovarian function and implantation parameters in non-pregnant female rats. Oral administration of PFE at doses of 100 and 200 mg·kg^−1^ body weight significantly enhanced follicular development, as evidenced by increased counts of primordial and primary follicles. These outcomes mirrored the stimulatory effects of clomiphene citrate, a standard ovulation-inducing agent.

Corresponding increases in uterine weight and epithelial proliferation were also observed, suggesting estrogen receptor-mediated modulation of uterine tissue.

Hormonal assays revealed a decrease in serum follicle-stimulating hormone (FSH) levels and concomitant elevations in luteinizing hormone (LH) and estradiol, indicating potential activation of the hypothalamic–pituitary–gonadal (HPG) axis. However, administration of the highest tested dose (500 mg·kg^−1^) led to a significant increase in post-implantation loss, implying potential embryotoxic effects at elevated concentrations [173].

These findings emphasize the importance of identifying an optimal therapeutic window, as excessive stimulation may disrupt implantation and embryonic viability.

#### 6.6.2. Male Reproductive Effects

A subsequent study by the same group [155] explored the effects of PFE on male reproductive parameters over a 21-day period. Rats treated with 100, 250, or 500 mg·kg^−1^ of the extract demonstrated notable improvements in sperm count, motility, and morphological integrity. Histological examination of testicular tissues revealed well-preserved seminiferous tubule architecture at all doses, although mild vacuolization was detected at the highest dose.

Endocrinological analysis showed a dose-dependent decrease in serum testosterone levels, accompanied by elevated LH concentrations. This paradoxical profile may indicate disruption of Leydig cell steroidogenesis or compensatory upregulation of gonadotropin secretion due to altered negative feedback.

Despite this testosterone suppression, the improvements in spermatogenic indices indicate a favorable effect on spermatogenesis and sperm functionality [155].

#### 6.6.3. Phytoestrogenic Mechanisms and Broader Context

The reproductive effects of *P. fruticosa* are likely mediated in part by its phytoestrogenic constituents, including flavonoids and triterpenoid saponins, which may bind to estrogen receptors and modulate gene expression along the HPG axis. Similar estrogenic mechanisms have been implicated in other phytoestrogen-rich botanicals used for treating menstrual irregularities, polycystic ovary syndrome (PCOS), and premature ovarian failure [172].

Furthermore, the adaptogenic and antioxidant properties of *P. fruticosa* may contribute to improved reproductive outcomes under stress conditions, oxidative load, or environmental insults. Its saponin profile shares pharmacodynamic similarities with ginsenosides from *Panax ginseng*, which are known to promote male sexual performance and regulate female endocrine homeostasis [172,174].

Of note, comparative analyses of triterpenoid structures support the hypothesis of shared receptor targets across Araliaceae-derived adaptogens.

Taken together, these findings position *P. fruticosa* as a promising multifunctional agent for reproductive health. Nonetheless, further investigations—including long-term toxicity, fertility, and teratogenicity studies—are essential to validate its safety and effectiveness in clinical use. The standardization of extract composition and dose–response characterization remain critical future directions.

### 6.7. Anti-Osteoclastogenic Potential of Polyscias fruticosa in Bone Health

The maintenance of skeletal homeostasis relies on a delicate balance between bone-forming osteoblasts and bone-resorbing osteoclasts. Disruption of this balance in favor of osteoclastic bone resorption underlies the pathogenesis of several skeletal disorders, including osteoporosis, rheumatoid arthritis, and Paget’s disease [175].

Osteoclasts originate from monocyte/macrophage precursors and undergo differentiation in response to key cytokines—macrophage colony-stimulating factor (M-CSF) and receptor activator of nuclear factor kappa-B ligand (RANKL). Engagement of RANKL with its receptor RANK activates intracellular cascades, such as the mitogen-activated protein kinase (MAPK) pathways and nuclear factor kappa-light-chain-enhancer of activated B cells (NF-κB), which in turn upregulate essential transcription factors, including c-Fos and NFATc1, driving osteoclastogenesis [176].

Tran et al. [19] investigated the anti-osteoclastogenic effects of ethanol extract from *P. fruticosa* leaves (EEPL) in both bone marrow-derived macrophages (BMMs) and RAW264.7 murine macrophage cells. Treatment with EEPL at concentrations ranging from 12.5 to 100 μg·mL^−1^ markedly inhibited RANKL-induced osteoclast differentiation in a dose-dependent manner. At the highest tested concentration (100 μg·mL^−1^), the extract suppressed the formation of tartrate-resistant acid phosphatase-positive (TRAP^+^) multinucleated cells by more than 85% relative to the RANKL-only control (*p* < 0.001).

EEPL also disrupted actin ring formation and significantly reduced the resorption pit area, indicative of impaired osteoclast function (*p* < 0.01 and *p* < 0.001 for higher doses).

This suppression of both osteoclastogenesis and resorptive capacity indicates functional inhibition beyond mere cytotoxicity.

These functional outcomes were accompanied by decreased mRNA and protein expression of osteoclast-associated markers, including TRAP, matrix metallopeptidase-9 (MMP-9), cathepsin K (CtsK), c-Fos, and NFATc1. Mechanistically, EEPL selectively inhibited the phosphorylation of p38 and JNK MAPKs while leaving ERK1/2 signaling unaffected, suggesting targeted interference with RANKL-mediated downstream signaling events [19,176].

The antiresorptive activity of *P. fruticosa* is comparable to that of other phytotherapeutics with known bone-protective properties. For example, ethanol extracts of *Osmanthus fragrans* have been shown to inhibit osteoclastogenesis by reducing intracellular reactive oxygen species and attenuating both NF-κB and MAPK signaling pathways [176].

Likewise, *Perilla frutescens* hexane leaf extract inhibited osteoclast differentiation through suppression of MMP-9 expression and RANKL pathway modulation in both cellular and animal models [177].

Such comparisons underscore the growing relevance of botanicals in bone health management, particularly those modulating osteoimmune crosstalk.

Given the adverse effects associated with conventional antiresorptive agents such as bisphosphonates, including osteonecrosis of the jaw and atypical femoral fractures, plant-based therapeutics like EEPL offer an attractive, multitarget alternative with a favorable safety profile.

Notably, *P. fruticosa* exhibits a broad pharmacological spectrum encompassing anti-inflammatory, antioxidant, neuroprotective, antidiabetic, and osteoprotective activities. The multitarget profile of *P. fruticosa* suggests potential benefits in complex pathologies such as postmenopausal osteoporosis, where inflammation, oxidative stress, and hormonal dysregulation coexist.

Its principal active compounds, triterpenoid saponins (e.g., PFS), flavonoids, and polyacetylenes, are implicated in these diverse biological effects.

In particular, saponins may modulate RANKL-induced transcription, while polyacetylenes could contribute to anti-inflammatory signaling inhibition.

However, challenges remain, particularly concerning the limited oral bioavailability of saponins and the potential for endocrine modulation at high doses. Further research, including in vivo validation in osteoporotic models, pharmacokinetic and toxicological profiling, and the development of targeted delivery systems such as nanocarriers, is necessary to fully elucidate and harness the anti-osteoclastogenic potential of *P. fruticosa* in clinical bone health management.

Moreover, standardization of extract composition and identification of active constituents are critical steps toward therapeutic development.

### 6.8. Author’s Perspective on the Pharmacological Potential of Polyscias fruticosa

*Polyscias fruticosa* emerges as a pharmacologically versatile medicinal plant whose broad spectrum of biological activities—ranging from antidiabetic and anti-inflammatory to neuroprotective, antimicrobial, and osteoprotective—has been increasingly substantiated by preclinical evidence [10,11,13,15,17,18,19,20,21]. Unlike many botanical agents that exert isolated effects, *P. fruticosa* demonstrates a multitarget pharmacodynamic profile, largely attributed to its diverse phytochemical composition, including triterpenoid saponins, polyacetylenes, flavonoids, phenolic acids, and sterols [3,4,6,10,11,16].

A critical comparative analysis with related medicinal plants—such as *Panax ginseng*, *Morus alba*, and *Momordica charantia*—highlights *P. fruticosa*’s unique advantages, including dual enzyme inhibition in diabetes management, mitochondrial stabilization in neurodegeneration, and superior antibacterial activity due to polyacetylene-rich fractions [10,11]. Furthermore, its adaptogenic and immunomodulatory properties provide a theoretical basis for its use in hormone-sensitive conditions, such as reproductive disorders and postmenopausal osteoporosis.

However, despite these promising findings, significant knowledge gaps remain. First, many pharmacological studies rely on ethanolic or crude extracts without standardization, making reproducibility and dose extrapolation challenging. Second, the *oral bioavailability and pharmacokinetics* of key compounds, particularly saponins, remain poorly defined. Third, safety data from long-term and reproductive toxicity studies are scarce—particularly in relation to embryotoxicity observed at high doses in female rats [173].

Importantly, the recent application of *P. fruticosa*-derived phytochemicals in green synthesis of metal-based nanoparticles opens new avenues for targeted anticancer therapy [169,170]. Nevertheless, this nanomedical potential demands further optimization of formulation strategies, cytotoxicity profiling in non-cancerous cells, and in vivo validation.

To advance *P. fruticosa* toward evidence-based clinical application, the following priorities are recommended:

- Standardization and quantification of active fractions;
- Comparative efficacy studies in validated animal models;
- Mechanistic investigations using omics platforms;
- Toxicological profiling under chronic and reproductive exposure;
- Development of delivery systems to overcome bioavailability limitations.

In summary, *P. fruticosa* represents a phytotherapeutic candidate with strong applicative potential. Its complex pharmacological potential aligns with the multifactorial nature of chronic diseases, but systematic research efforts—both mechanistic and clinical—are required to unlock its full therapeutic promise.

## 7. Conclusions and Future Perspectives

*Polyscias fruticosa* emerges as a promising, yet underutilized, medicinal plant situated at the intersection of plant biotechnology, phytochemistry, and pharmacology. Its demonstrated amenability to in vitro propagation, structurally diverse array of secondary metabolites, and broad spectrum of pharmacological activities underscore its value as a candidate for phytotherapeutic and nutraceutical development.

In plant biotechnology, robust micropropagation protocols employing apical meristem cultures and somatic embryogenesis have been successfully established. Suspension cultures derived from callus tissues have demonstrated biosynthetic competence for various classes of secondary metabolites, including triterpenoid saponins and phenolic compounds. However, current investigations remain limited to the laboratory scale. Future efforts should prioritize the upscaling of biomass and metabolite production using semi-technical or industrial bioreactor systems. Optimization of culture media, the application of elicitors, control of physicochemical parameters, and the implementation of AI-assisted modeling of growth and metabolite dynamics represent key directions for future research. Notably, the absence of studies on genetically transformed root cultures in *P. fruticosa* presents an opportunity for the development of alternative, non-transgenic production platforms aligned with sustainable biotechnological standards.

Phytochemically, *P. fruticosa* exhibits a complex profile comprising triterpenoid saponins, flavonoids, phenolic acids, sterols, polyacetylenes, and fatty acid derivatives. Many of these compounds share structural similarities with ginsenosides and other metabolites characteristic of the Araliaceae family. However, further elucidation of stereochemical configurations and biosynthetic pathways remains a critical gap. Integrative metabolomic approaches combining UPLC-QTOF-MS/MS, GC×GC-MS, and NMR spectroscopy with transcriptomic profiling are essential to deepen our understanding of its metabolic landscape. In particular, the identification and functional characterization of key biosynthetic enzymes—such as cytochrome P450s, glycosyltransferases, and methyltransferases—would facilitate targeted metabolic engineering for enhanced compound production.

From a pharmacological standpoint, *P. fruticosa* has demonstrated anti-inflammatory, antidiabetic, antiasthmatic, neuroprotective, and antiresorptive activities in preclinical models. Nonetheless, most studies remain exploratory and lack comprehensive mechanistic and translational validation. Future research should incorporate systems pharmacology approaches, including molecular docking, multi-omics target validation, and disease-specific in vivo models. The pharmacodynamic interplay between compound classes, such as saponin–flavonoid synergy, should be investigated to uncover pleiotropic effects on interconnected signaling networks. Standardized extracts and purified fractions must be subjected to rigorous pharmacokinetic and toxicological assessments, followed by well-designed clinical trials, particularly in chronic metabolic and neuroinflammatory disorders.

In conclusion, *P. fruticosa* exemplifies a next-generation medicinal plant with strong translational potential. Its advancement demands a multidisciplinary framework integrating innovative plant tissue culture technologies, state-of-the-art analytical chemistry, and mechanistically driven pharmacological research. As an alternative source of Araliaceae-type saponins, *P. fruticosa* holds particular promise as a sustainable, bioactive reservoir for future phytopharmaceutical applications.

## Figures and Tables

**Table 1:** Summary of triterpenoid glycosides isolated from roots, leaves, and in vitro cultures of *P. fruticosa*.

No.	Trivial Name	Systematic Name	Molecular Formula	Molecular Weight [g/mol]	Plant Source	Identification Method	Ref.
1	-	3-O-[*β*-D-galactopyranosyl-(1→2)-*β*-D-glucuronopyranosyl] oleanolic acid	C_42_H_66_O_14_	794.44	Leaves	FAB, MS (negative)	[69]
2	-	3-O-[*α*-L-rhamnopyranosyl-(1→4)-*β*-D-glucuronopyranosyl]28-O-*β*-D-glucopyranosyl oleanolic acid	C_48_H_76_O_19_	940	Leaves (yellow variety)	FAB, MS (negative)	[5]
3	Polyscioside A	3-O-(β-D-glucopyranosyl-(1→2)-β-D-glucopyranosyl-(1→4)-β-D-glucuronopyranosyl) oleanolic acid	C_48_H_76_O_19_	956.49	Leaves, roots	HR FAB-MS (negative), NMR	[3]
4	Polyscioside B	3-O-[*α*-L-arabinopyranosyl-(1→4)-*β*-D-glucopyranosyl-(1→2)-*β*-D-glucuronopyranosyl] oleanolic acid	C_47_H_74_O_18_	926.47	Roots	HR FAB-MS (negative), NMR	[3]
5	Polyscioside C	3-O-[*β*-D-galactopyranosyl-(1→2)-*β*-D-glucopyranosyl-(1→3)-*β*-D-glucuronopyranosyl] oleanolic acid	C_48_H_76_O_19_	956.49	Roots	HR-FAB-MS (negative), NMR	[3]
6	Polyscioside D	3-O-[*β*-D-glucopyranosyl-(1→2)-[*β*-D-glucopyranosyl-(1→4)]-*β*-D-glucuronopyranosyl]- oleanolic acid 28-O-*β*-D-glucopyranosyl ester	C_54_H_86_O_24_	1118.54	Leaves, roots	HR FAB-MS (negative), NMR	[3]
7	Polyscioside E	3-O-[*α*-L-arabinopyranosyl-(1→4)-[β-D-glucopyranosyl-(1→2)]-*β*-D-glucuronopyranosyl]oleanolic acid 28-O-*β*-D-glucopyranosyl ester	C_53_H_84_O_23_	1088.53	Roots	HR FAB-MS (negative), NMR	[3]
8	Polyscioside F	3-O-[*β*-D-galactopyranosyl-(1→2)-[*β*-D-glucopyranosyl-(1→3)]-*β*-D-glucuronopyranosyl] oleanolic acid 28-O-*β*-D-glucopyranosyl ester	C_54_H_86_O_24_	1118.54	Leaves	HR FAB-MS (negative), NMR	[3]
9	Polyscioside G	3-O-[*β*-D-glucopyranosyl-(1→4)-*β*-D-glucuronopyranosyl]oleanolic acid 28-O-α-L-rhamnopyranosyl-(1→3)-*β*-D-glucopyranosyl ester	C_54_H_86_O_23_	1102.55	Leaves	HR FAB-MS (negative), NMR	[3]
10	Polyscioside H	3-O-[*β*-D-glucopyranosyl-(1→4)-*β*-D-glucopyranosyl-(1→2)-*β*-D-glucuronopyranosyl] oleanolic acid 28-O-α-L-rhamnopyranosyl-(1→3)-*β*-D-glucopyranosyl ester	C_60_H_96_O_28_	1264.65	Leaves	HR FAB-MS (negative), NMR	[3]
11	Ladyginoside A (calendulaglycoside E)	3-*O*-[*β*-D-glucopyranosyl (1-4)-*β*-D-glucuronopyranosyl] oleanolic acid	C_42_H_66_O_14_	794.44	Leaves, cell suspension culture	UPLC-ESI-MS	[3]
12	Zingibroside R1	3-*O*-[*β*-D-glucopyranosyl (1-2)-*β*-D-glucuronopyranosyl] oleanolic acid	C_42_H_66_O_14_	794.44	Leaves	NMR, MS	[3]
13	Saponin PFS	3-O-[*β*-D-glucopyranosyl-(1→4)-*β*-D-glucuronopyranosyl] oleanolic acid 28-O-*β*-D-glucopyranosyl ester	C_48_H_76_O_19_	956.49	Leaves, root, cell suspension culture	HR-ESI-MS (negative)	[3,57]
14	-	oleanolic acid-28-O-*β*-D-glucopyranosyl-D-glucopyranosyl-rhamnopyranoside		926	Leaves, roots	ESI-MS, NMR	[6]
15	-	3-O-*α*-L-arabinopyranosyl-oleanolic acid-28-O-*β*-D-glucopyranosyl-D-glucopyranosyl-rhamnopyranoside		1058	Leaves, roots	ESI-MS, NMR	[6]
16	Polyscioside I	3-O-[*β*-D-glucopyranosyl-(1→2)-[*β*-D-glucopyranosyl-(1→4)]-*β*-D-glucuronopyranosyl] oleanolic acid 28-O-*β*-D-glucopyranosyl-(1→2)-*β*-D-galactopyranosyl ester	C_60_H_97_O_29_	1280.61	Leaves	HR ESI-MS, NMR	[14]
17	Polyscioside J	3-O-[*β*-D-glucopyranosyl-(1→4)]-β-D-(6-O-methyl)glucuronopyranosyl] oleanolic acid 28-O-*β*-D-glucopyranosyl ester	C_49_H_78_O_19_	970.50	Leaves	HR ESI-MS (negative)	[7]
18	Polyscioside K	3-O-[*β*-D-glucopyranosyl-(1→4), β-D-glucopyranosyl-(1→2)]-*β*-D-(6-O-methyl)glucuronopyranosyl] oleanolic acid	C_49_H_78_O_19_	970.50	Leaves	HR-ESI-MS (negative)	[7]
19	Chikusetsusaponin IVa	3-*O*-[*β*-D-glucuronopyranosyl] oleanolic acid 28-*O*-*β*-D-glucopyranosyl ester	C_42_H_66_O_14_	794.44	Leaves	NMR, MS	[7]

HR FAB-MS—high-resolution fast atom bombardment mass spectrometry; NMR—nuclear magnetic resonance.

**Table 2:** Phenolic compounds identified in *Polyscias fruticosa*: structural characteristics, plant sources, and extraction methods.

No.	Compound Name	Compound Class	Molecular Formula	Exact Mass	Plant Source	Extraction Method	Ref.
20	Kaempferol-3-rhamnoside/afzelin/kaempferol-3-O-alpha-L-rhamnoside	Flavonoids	C_21_H_20_O_10_	432.10	Leaves	EtOH (95%), maceration 72 h, 25 °C, EtOAc/n-hexane fraction	[8]
21	Quercetin-3-rhamnoside/quercitrin/quercetin 3-L-rhamnoside	Flavonoids	C_21_H_20_O_11_	448.10	Leaves	EtOH (95%), maceration 72 h, 25 °C, EtOAc/n-hexane fraction	[8]
22	Esculetin/ aesculetin	Phenolic compounds	C_9_H_6_O_4_	286.24	Leaves	EtOH (95%), maceration 72 h, 25 °C, EtOAc/n-hexane fraction	[8]
23	Liquiritigenin/ 7-hydroxy-2-(4-hydroxy-phenyl)-chroman-4-one	Flavanones	C_15_H_12_O_4_	256.07	Leaves	EtOH (95%), maceration 72 h, 25 °C, EtOAc/n-hexane fraction	[8]
24	(*E*)-3,4-dihydroxycinnamic Acid/ caffeic acid	Phenolic acids	C_9_H_8_O_4_	180.04	Leaves	EtOH (95%), maceration 72 h, 25 °C, EtOAc/n-hexane fraction	[8]
25	4-hydroxycinnamaldehyde	Phenolic compounds	C_9_H_8_O_2_	148.05	Leaves	EtOH (95%), maceration 72 h, 25 °C, EtOAc/n-hexane fraction	[8]
26	(*E*)-isoferulaldehyde/ 3-hydroxy-4-methoxycinnamaldehyde	Phenolic compounds	C_10_H_10_O_3_	178.06	Leaves	EtOH (95%), maceration 72 h, 25 °C, EtOAc/n-hexane fraction	[8]
27	Protocatechuic acid/ 3,4-dihydroxybenzoic acid	Phenolic acids	C_7_H_6_O_4_	154.02	Roots	EtOH 95%, maceration at RT Et_2_O fraction	[21]
28	Politoic acid/ (E)-2-(4-((E)-2-carboxyvinyl)-2-methoxyphenoxy)-3-(4-hydroxyphenyl)acrylic acid	Phenolic acids	C_18_H_16_O_7_	344.32	Roots	EtOH 95%, maceration at RT, Et_2_O fraction	[21]
29	8-O-4-dehydrodiferulic acid	Phenolic acids	C_20_H_18_O_8_	386.35	Roots	EtOH 95%, maceration at RT, Et_2_O fraction	[21]
30	8-O-4/8-O-4-dehydrotriferulic acid	Phenolic acids	C_30_H_26_O_12_	578.52	Roots	EtOH 95%, maceration at RT, Et_2_O fraction	[21]
31	1′-hydroxyeugenol/ 4-(1-hydroxy-allyl)-2-methoxy-phenol	Polyphenols	C_10_H_12_O_3_	180.07	Leaves	UAE EtOH (95%), n-hexane/DCM (PFLD) 1 h, 30 °C	[10]
32	2,6-dimethoxy-4-vinylphenol/ 4-ethenyl-2,6-dimethoxyphenol	Polyphenols	C_10_H_12_O_3_	180.07	Leaves	UAE EtOH (95%), n-hexane/DCM (PFLD) 1 h, 30 °C	[10]
33	2,6-dimethoxyphenol/ syringol/ pyrogallol 1,3-dimethyl ether	Polyphenols	C_8_H_10_O_3_	154.06	Leaves	UAE EtOH (95%), n-hexane/DCM (PFLD) 1 h, 30 °C)	[10]
34	4-vinylphenol/ 4-hydroxystyrene/	Polyphenols	C_8_H_8_O	120.05	Leaves	UAE EtOH (95%), n-hexane/DCM (PFLD) 1 h, 30 °C	[10]
35	vanillin/ 4-hydroxy-3-methoxybenzaldehyde/ vanillaldehyde	Polyphenols	C_8_H_8_O_3_	152.04	Leaves	UAE EtOH (95%), n-hexane/DCM (PFLD) 1 h, 30 °C	[10]
36	2-methoxy-4-vinylphenol/ 4-vinylguaiacol/ p-vinylguaiacol	Polyphenols	C_9_H_10_O_2_	150.06	Leaves	UAE EtOH (95%), n-hexane/DCM (PFLD) 1 h, 30 °C	[10]
37	4-allyl-2,6-dimethoxyphenol/ methoxyeugenol/ 4-allylsyringol	Polyphenols	C_11_H_14_O_3_	194.09	Leaves	UAE EtOH (95%), n-hexane/DCM (PFLD) 1 h, 30 °C	[10]
38	1,2-bis(4-hydroxy-3-methoxyphenyl)ethylene	Polyphenols	C_16_H_16_O_4_	272.10	Leaves	UAE EtOH (95%), n-hexane/DCM (PFLD) 1 h, 30 °C	[10]
39	Sinapyl alcohol/ 4-(3-hydroxyprop-1-en-1-yl)-2,6-dimethoxyphenol	Phenylpropanoid (lignin precursor)	C_11_H_14_O_4_	210.08	Leaves	UAE EtOH (95%), n-hexane/DCM (PFLD) 1 h, 30 °C	[10]

EtOH (95%)—95% ethanol extraction, Et_2_O—diethyl ether fraction (liquid–liquid partitioning with diethyl ether), DCM (PFLD)—dichloromethane fraction (*Polyscias fruticosa* leaf dichloromethane fraction), EEPF (EtOAc fraction)—ethyl acetate fraction of ethanol extract from *P. fruticosa* roots; UAE, n-hexane—ultrasound-assisted extraction using n-hexane at 30 °C for 1 h [10]; maceration, EtOH (95%), 72 h, 25 °C—ethanol maceration at room temperature [8].

**Table 3:** Sterols and steroidal derivatives identified in *Polyscias fruticosa*: molecular characteristics, extraction conditions, and analytical methods.

No.	Compound Name	Type	Molecular Formula	Plant Source	Extraction Method	Identification Method	Ref.
40	Stigmasterol	Phytosterol	C_29_H_48_O	leaves	UAE EtOH (95%), n-hexane/DCM (PFLD) 1 h, 30 °C	GC-MS	[4,10]
41	(3β,5α)-Stigmasta-7,16-dien-3-ol	Sterol derivative	C_29_H_48_O	leaves	UAE EtOH (95%), n-hexane/DCM (PFLD) 1 h, 30 °C	GC-MS	[10]
42	22-dehydro-24-isopropylcholesterol	Atypical sterol	C_30_H_48_O	roots	Petroleum ether/diethyl ether (1:1)	X-ray crystallography	[118]

DCM extraction—dichloromethane extraction, EtOH (95%)—extraction using 95% ethanol; petroleum ether/diethyl ether (1:1)—extraction with a mixture of petroleum ether and diethyl ether (1:1, *v*/*v*); GC-MS—gas chromatography–mass spectrometry, X-ray crystallography—X-ray diffraction-based structural analysis. Identification of compounds 40 and 41 by GC-MS was based on comparison with the NIST spectral library and published reference data [10]. Chromatographic parameters: Rxi-5MS column (30 m × 0.25 mm i.d., 0.25 μm), helium carrier gas (1.12 mL/min), 1 µL injection (split ratio 1:20), injector temperature 280 °C, oven temperature 50–280 °C (10 °C/min), EI mode (70 eV), scan range *m*/*z* 50–600.

**Table 4:** Volatile and lipophilic constituents of *Polyscias fruticosa* identified by GC-MS and other analytical methods.

No.	Compound Name	Class of Compounds	Molecular Formula	Plant Source	Identification Method	Ref.
43	Limonene	Monoterpene	C_10_H_16_	Leaves	GC-MS	[9]
44	(Z)-Hex-3-en-1-yl acetate	Monoterpene	C_8_H_14_O_2_	Leaves	GC-MS	[9]
45	1-hexanol	Monoterpene	C_6_H_14_O	Leaves	GC-MS	[9]
46	(Z)-Hex-3-en-l-ol	Monoterpenoid	C_6_H_12_ O	Leaves	GC-MS	[9]
47	3-bornanone oxime	Oxygenated Monoterpenes	C_10_H_17_NO	Leaves	GC-MS	[10]
48	Actinidiolide	Norisoprenoid lactone	C_11_H_16_O_2_	Leaves	GC-MS	[10]
49	Loliolide	Norisoprenoid lactone	C_11_H_16_O_3_	Leaves	GC-MS	[10]
50	^®^-1-(7,7-dimethyl-1,3,4,5,6,7-hexahydroisobenzofuran-1α-yl)-2-propanol	Monoterpenoid derivative (furan)	C_13_H_22_O_2_	Leaves	GC-MS	[10]
51	(E)-γ-bisabolene	Sesquiterpene	C_15_H_24_	Leaves	GC-MS	[9]
52	α-bergamotene	Sesquiterpene	C_15_H_24_	Leaves	GC-MS	[9,10]
53	α-cubebene	Sesquiterpene	C_15_H_24_	Leaves	GC-MS	[9]
54	α-ylangene	Sesquiterpene	C_15_H_24_	Leaves	GC-MS	[9,10]
55	Germacrene D	Sesquiterpene	C_15_H_24_	Leaves	GC-MS	[9,10]
56	Germacrene B	Sesquiterpene	C_15_H_24_	Leaves	GC-MS	[9]
57	β-caryphyllene	Sesquiterpene	C_15_H_24_	Leaves	GC-MS	[9]
58	β-elemene	Sesquiterpene	C_15_H_24_	Leaves	GC-MS	[9]
59	δ-elemene	Sesquiterpene	C_15_H_24_	Leaves	GC-MS	[9]
60	γ-elemene	Sesquiterpene	C_15_H_24_	Leaves	GC-MS	[9]
61	α-copaene	Sesquiterpene	C_15_H_24_	Leaves	GC-MS	[9,10]
62	Aromadedrene	Sesquiterpene	C_15_H_24_	Leaves	GC-MS	[9,10]
63	α-humulene	Sesquiterpene	C_15_H_24_	Leaves	GC-MS	[9]
64	δ-cadinene	Sesquiterpene	C_15_H_24_	Leaves	GC-MS	[9]
65	α-ylangene	Sesquiterpene	C_15_H_24_	Leaves	GC-MS	[9,10]
66	α-copaene	Sesquiterpene	C_15_H_24_	Leaves	GC-MS	[9,10]
67	β-bourbonene	Sesquiterpene	C_15_H_24_	Leaves	GC-MS	[9]
68	β-cubebene	Sesquiterpene	C_15_H_24_	Leaves	GC-MS	[9]
69	β-gurjunene	Sesquiterpene	C_15_H_24_	Leaves	GC-MS	[9,10]
70	Calacorene	Sesquiterpene	C_15_H_24_	Leaves	GC-MS	[9]
71	γ-cadinene	Sesquiterpene	C_15_H_24_	Leaves	GC-MS	[10]
72	Caryophyllene oxide	Oxygenated sesquiterpene	C_15_H_24_O	Leaves	GC-MS	[10]
73	Calamenene	Oxygenated sesquiterpene	C_15_H_22_O	Leaves	GC-MS	[9]
74	Alismol	Oxygenated sesquiterpene	C_15_H_26_O	Leaves	GC-MS	[10]
75	Opposita-4(15),7(11)-dien-1β-ol	Oxygenated sesquiterpene	C_15_H_24_O	Leaves	GC-MS	[10]
76	Alloisolongifolene alcohol	Oxygenated sesquiterpene	C_15_H_24_O	Leaves	GC-MS	[10]
77	(4S,5R)-5-hydroxycaryophyll-8(13)-ene-4,12-epoxide	Oxygenated sesquiterpene	C_15_H_24_O_2_	Leaves	GC-MS	[10]
78	Alismoxide	Oxygenated sesquiterpene	C_15_H_24_O_2_	Leaves	GC-MS	[10]
79	β-Oplopenone	Oxygenated sesquiterpene	C_15_H_22_O	Leaves	GC-MS	[10]
80	Neoclovene oxide	Oxygenated sesquiterpene	C_15_H_22_O	Leaves	GC-MS	[10]
81	(−)-isolongifolol methyl ether	Oxygenated sesquiterpene (ether)	C_16_H_28_O	Leaves	GC-MS	[10]
82	Ledol	Oxygenated sesquiterpene	C_15_H_22_O	Leaves	GC-MS	[10]
83	Caryophyllenol II	Oxygenated sesquiterpene	C_15_H_24_O	Leaves	GC-MS	[10]
84	6,10,14-trimethylpentadecan-2-one	Aliphatic ketone	C_18_H_36_O	Leaves	GC-MS	[10]
85	Neophytadiene	Diterpenoid hydrocarbon	C_20_H_38_	Leaves	GC-MS	[10]
86	Phytol	Diterpenoid alcohol	C_20_H_40_O	Leaves	GC-MS	[10]
87	(Z)-1,3-Phytadiene	acyclic diterpene	C_20_H_38_	Leaves	GC-MS	[10]

GS-MS—gas chromatography–mass spectrometry. All volatile and lipophilic compounds listed in Table 4 were identified using GC-MS. For the majority of samples, analyses were performed on an Rxi-5MS capillary column (30 m × 0.25 mm i.d., 0.25 μm film thickness) with helium as carrier gas (1.12 mL/min), injector temperature 280 °C, injection volume 1 μL (split 1:20), and oven program from 50 °C (3 min hold) to 280 °C (10 min hold) at 10 °C/min. Mass spectra were recorded in EI mode at 70 eV (scan range *m*/*z* 50–600). Earlier GC-MS identifications (e.g., Brophy et al. [9]) did not report full chromatographic parameters. Biological activity data are discussed in Section 5.4 and Section 5.5. Only compounds with documented or presumed pharmacological significance are addressed in the discussion.

**Table 5:** Tocopherols identified in *Polyscias fruticosa* leaf extracts by GC-MS.

No.	Compound Name	Class of Compound	Molecular Formula	Plant Source	Identification Method	Ref.
88	δ-tocopherol	Tocol (Vitamin E)	C_27_H_46_O_2_	Leaves	GC-MS	[10]
89	β-tocopherol	Tocol (Vitamin E)	C_28_H_48_O_2_	Leaves	GC-MS	[10]
90	DL-α-tocopherol	Tocol (Vitamin E)	C_29_H_50_O_2_	Leaves	GC-MS	[10]
91	α-tocopherol	Tocol (Vitamin E)	C_29_H_50_O_2_	Leaves	GC-MS	[10]

GC-MS—gas chromatography–mass spectrometry. Compounds were identified using the following conditions: Rxi-5MS capillary column (30 m × 0.25 mm i.d., 0.25 μm film), helium as carrier gas (1.12 mL/min), injector at 280 °C, split 1:20, injection volume 1 μL. Oven program: 50–280 °C at 10 °C/min (3 min initial, 10 min final hold). EI at 70 eV, scan range *m*/*z* 50–600 [10]. The biological functions of tocopherols are discussed in Section 5.6.

**Table 6:** Polyacetylenes identified in *Polyscias fruticosa*, including compound class, plant source, and analytical methods.

No.	Compound Name	Class of Compounds	Molecular Formula	Plant Source	Identification Method	Ref.
92	Falcarinol (panaxynol)	C17 polyacetylene; monoacetylene alcohol	C_17_H_24_O	Roots, leaves	GC-MS, NMR	[4,10,11]
93	Heptadeca-1,8- diene-4,6-diyne- 3-ol-10-one	C17 polyacetylene; diacetylene diol	C_17_H_22_O_2_	Roots, leaves	GC-MS, NMR	[4,11]
94	Heptadeca-1,8-diene-4,6-diyne-3,10-diol/ (seselidiol)	C17 polyacetylene; diacetylene alcohol-ketone	C_17_H_24_O_2_	Roots, leaves	GC-MS, NMR	[4,11]
95	Hexadeca-5,7,9,11-tetrayne-1,16-diol	C17 polyacetylene; polyacetylene diol	C_16_H_18_O_2_	Leaves	GC-MS	[10]

GC-MS—gas chromatography–mass spectrometry. NMR—nuclear magnetic resonance. All compounds were identified based on GC-MS analysis and comparison with literature data [4,10,11]. Their biological activities are discussed in detail in Section 5.7.

**Table 7:** Fatty acid derivatives identified in *Polyscias fruticosa* by GC-MS and their classification.

No.	Compound Name	Type	Molecular Formula	Molecular Weight (g/mol)	Plant Source	Identification Method	Ref.
96	9,11-octadecadienoic acid	CLA isomer	C_18_H_32_O_2_	280.24	Leaves	GC-MS	[10]
97	10-trans,12-cis-linoleic acid	CLA isomer	C_18_H_32_O_2_	280.24	Leaves	GC-MS	[10]
98	2-linoleoylglycerol	Monoacylglycerol (MAG)	C_21_H_38_O_4_	354.27	Leaves	GC-MS	[10]
99	Linoleic acid	Polyunsaturated fatty acid (PUFA)	C_18_H_32_O_2_	280.24	Leaves	GC-MS	[10]
100	Linolenic acid	Polyunsaturated fatty acid (PUFA)	C_18_H_30_O_2_	278.22	Leaves	GC-MS	[10]
101	Lionoelaidic acid	Polyunsaturated fatty acid (PUFA)	C_18_H_30_O_2_		Leaves	GC-MS	[10]
102	Palmitic acid	Saturated fatty acid (SFA)	C_16_H_32_O_2_	256.24	Leaves	GC-MS	[10]
103	Stearic acid	Saturated fatty acid (SFA)	C_18_H_36_O_2_	284.27	Leaves	GC-MS	[10]
104	Methyl linoleate	Fatty acid methyl ester (FAME)	C_19_H_34_O_2_	294.25	Leaves	GC-MS	[10]
105	Ethyl palmitate	Fatty acid ethyl ester (FAEE)	C_18_H_36_O_2_	284.27	Leaves	GC-MS	[10]
106	Myristic acid	Saturated fatty acid (SFA)	C_14_H_28_O_2_	228.20	Leaves	GC-MS	[10]
107	Octanoic acid	Fatty acid ester	C_8_H_16_O_2_	144.12	Leaves	GC-MS	[10]
108	2-palmitoylglycerol	Monoacylglycerol (MAG)	C_19_H_38_O_4_	330.28	Leaves	GC-MS	[10]
109	Diisobutyl adipate	Aliphatic diester of a dicarboxylic acid	C_14_H_26_O_4_	298.51	Leaves	GC-MS	[10]

GC-MS—gas chromatography–mass spectrometry. All fatty acids and related compounds were identified using GC-MS under the same analytical conditions previously described (see footnote to Table 4). The biological activities of selected compounds are discussed in Section 5.8.

**Table 8:** Newly identified volatile and lipophilic constituents of *Polyscias fruticosa* detected by GC-MS.

No.	Compound Name	Type	Molecular Formula	Molecular Weight (g/mol)	Plant Source	Identification Method	Ref.
110	3-hydroxy-4,5-dimethylfuran-2(5H)-one	Furanone-type lactone (oxygenated heterocycle)	C_6_H_8_O_3_	128.05	Leaves	GC-MS	[10]
111	Octanal	Aliphatic aldehyde	C_8_H_16_O	128.12	Leaves	GC-MS	[10]
112	Hexadecanal	Long-chain saturated aliphatic aldehyde	C_16_H_32_O	240.25	Leaves	GC-MS	[10]
113	3-methylene-7,11-dimethyl-1-dodecene	Branched unsaturated hydrocarbon	C_15_H_28_	208.22	Leaves	GC-MS	[10]
114	Linoleyl alcohol	Long-chain unsaturated fatty alcohol	C_18_H_34_O_2_	266.26	Leaves	GC-MS	[10]

GC-MS—gas chromatography–mass spectrometry. Compounds (**110**–**114**) were identified using GC-MS under the same analytical conditions previously described (see footnote to Table 4). Their biological activities are discussed in Section 5.9.

**Table 9:** Structurally diverse metabolites newly identified in *Polyscias fruticosa* detected by GC-MS analysis.

No.	Compound Name	Type	Molecular Formula	Molecular Weight (g/mol)	Plant Source	Identification Method	Ref.
115	4-ethoxy-4-oxobutanoic acid	Carboxylic acid derivative (β-ketoester-type compound)	C_6_H_10_O_4_	146.06	Leaves	GC-MS	[10]
116	2,3-dihydrothiophene	Sulfur-containing heterocyclic compound	C_4_H_6_S	86.02	Leaves	GC-MS	[10]
117	Malic acid	Dicarboxylic acid (α-hydroxy acid)	C_4_H_6_O_5_	134.02	Leaves	GC-MS	[10]
118	Levoglucosan	Carbohydrate derivative (1,6-anhydro sugar)	C_6_H_10_O_5_	162.05	Leaves	GC-MS	[10]
119	4,3′-difluoro-4′-methoxybiphenyl	Halogenated aromatic hydrocarbon (fluoroalkylated biphenyl)	C_13_H_10_F_2_O	220.07	Leaves	GC-MS	[10]
120	Indole-3-carboxaldehyde	Indole derivative (aromatic heterocyclic aldehyde)	C_9_H_7_NO	145.05	Leaves	GC-MS	[10]
121	4,6,6-trimethyl-2-(3-methylbuta-1,3-dienyl)-3-oxatricyclo[5.1.0.0(2,4)]octane	Oxygenated polycyclic hydrocarbon (tricyclic ether–terpenoid-like compound)	C_15_H_22_O	218.16	Leaves	GC-MS	[10]

GC-MS—gas chromatography–mass spectrometry. Compounds (**115**–**121**) were identified using GC-MS under the same analytical conditions previously described (see footnote to Table 4). Their biological activities are discussed in Section 5.10.

## Data Availability

Data are contained within the article.
